# Multimodal multitask learning for predicting MCI to AD conversion using stacked polynomial attention network and adaptive exponential decay

**DOI:** 10.1038/s41598-023-37500-7

**Published:** 2023-07-11

**Authors:** Ngoc-Huynh Ho, Yang-Hyung Jeong, Jahae Kim

**Affiliations:** 1 Department of Artificial Intelligence Convergence, Chonnam National University, Gwangju, 61186 South Korea; 2 Department of Nuclear Medicine, Chonnam National University Hospital, Gwangju, 61469 South Korea

**Keywords:** Medical research, Electrical and electronic engineering

## Abstract

Early identification and treatment of moderate cognitive impairment (MCI) can halt or postpone Alzheimer’s disease (AD) and preserve brain function. For prompt diagnosis and AD reversal, precise prediction in the early and late phases of MCI is essential. This research investigates multimodal framework-based multitask learning in the following situations: (1) Differentiating early mild cognitive impairment (eMCI) from late MCI and (2) predicting when an MCI patient would acquire AD. Clinical data and two radiomics features on three brain areas deduced from magnetic resonance imaging were investigated (MRI). We proposed an attention-based module, Stack Polynomial Attention Network (SPAN), to firmly encode clinical and radiomics data input characteristics for successful representation from a small dataset. To improve multimodal data learning, we computed a potent factor using adaptive exponential decay (AED). We used experiments from the Alzheimer’s Disease Neuroimaging Initiative (ADNI) cohort study, which included 249 eMCI and 427 lMCI participants at baseline visits. The proposed multimodal strategy yielded the best c-index score in time prediction of MCI to AD conversion (0.85) and the best accuracy in MCI-stage categorization ($83.19\%$). Moreover, our performance was equivalent to that of contemporary research.

## Introduction

Memory loss and cognitive decline are clinical symptoms of Alzheimer’s disease (AD)^1^, which is a progressive neurodegenerative sickness that affects the brain. It is the most prevalent cause of dementia in individuals over the age of 65. In 2021, over 55 million people live with dementia worldwide^2^. AD accounts for 60 to 80 percent of all dementia cases according to reports^3^. AD is defined by the accumulation of beta-amyloid and tau proteins in the brain^4^, which hinder normal cognitive activities. This often emerges as alterations in memory, analytical thinking, perception, language, mood, and emotions, and eventually impairs physical control over the body. Several studies have been conducted in recent years in an attempt to identify early biomarkers that may be used to assess Alzheimer’s disease risk prior to the onset of symptoms in a speedy and thorough manner^5–7^.

Currently, mild cognitive impairment (MCI), which is a prodromal stage of Alzheimer’s disease (AD), has attracted much attention because of the high likelihood that it will advance to AD. MCI is characterized by a minor but perceptible and quantifiable reduction in cognitive ability, including memory and reasoning abilities. An individual diagnosed with MCI may be at risk of acquiring Alzheimer’s disease in the future, or the condition may be due to age-related cognitive deterioration, underscoring the necessity of early detection of the condition. Research has shown that the risk of late MCI (lMCI) conversion to AD is higher than that of early MCI (eMCI)^8^. Identification of potentially sensitive diagnostic indicators that change in response to illness development may aid the physician in making a diagnosis. If detected early in the course of MCI, individuals can significantly lower their risk of developing AD by approximately one-third with rehabilitation activities and medication^9^. Regrettably, sensitive indicators vary according to disease development^10^, and there are presently no definite diagnostic biomarkers or viable therapies for Alzheimer’s disease^11^.

There is widespread agreement on the importance of and benefits of early detection of the condition. Numerous neurologists and medical researchers are currently devoting significant work to developing procedures for early diagnosis of AD, with consistently encouraging findings^12^. In recent decades, several studies have been proposed for automatic detection of AD^6,13–16^. Various neuroimaging signals such as magnetic resonance imaging (MRI)^17,18^, functional magnetic resonance imaging (fMRI)^19,20^, positron emission tomography (PET)^21,22^, electroencephalography (EEG)^23–25^, and magnetoencephalography (MEG)^26,27^ have been investigated to determine if there are any anomalous clustering coefficients or distinctive path lengths in the brain networks of AD patients. The ability to diagnose and categorize MCI at an early stage helps physicians to make better informed judgments about clinical intervention and treatment planning at a later stage, which has a significant influence on cost-effectiveness of long-term care services^28^. However, only a few studies on the features of brain networks in MCI patients have explored the properties of brain networks at different phases since brain abnormalities are so subtle^29,30^.

Feature fusion strategies have gained significant attention in the medical field for their ability to integrate diverse information sources and enhance diagnostic accuracy. Multimodal image fusion techniques, as highlighted by Wang et al.^31^, enable the combination of complementary information from different imaging modalities, such as MRI, CT, and PET, to improve disease interpretation. Deep learning-based approaches, exemplified by Li et al.^32^, leverage feature fusion to enhance medical diagnosis by effectively integrating multimodal information. Moreover, Tong et al.^33^ demonstrated the potential of feature fusion in Alzheimer’s disease diagnosis, utilizing hybrid weighted multiple kernel learning to integrate clinical assessments, genetic profiles, and neuroimaging data. By leveraging feature fusion strategies, medical researchers and practitioners can harness the power of multiple data modalities to improve disease detection, localization, and overall patient outcomes. In this paper, we will present a straightforward and efficient fusion equation designed to combine multimodal data.

In this paper, we proposed a novel attention-based mechanism for multimodal multitask learning of AD progression. We employed MRI scans and clinical data to distinguish eMCI from lMCI while also predicting the time to AD conversion. We extract three brain regions, in particular, such as gray matter (GM), white matter (WM), and Cerebrospinal Fluid (CSF) from T1-MRI image using the statistical parametric mapping (SPM) toolbox (https://www.fil.ion.ucl.ac.uk/spm/). Then, we estimated the texture and shape features from the masked regions using the PyRadiomics toolbox (https://pyradiomics.readthedocs.io/). Consecutively, we introduced a novel deep learning (DL) approach called stacked polynomial attention network (SPAN) for learning a more accurate approximation basis for all polynomials of bounded degree^34^. Two branches with SPAN and dropout layers are employed to encode the clinical and radiomics representations, and the prominent characteristics of both branches are effectively merged using our proposed formula, adaptive exponential decay (AED). The composite representation is scaled using a series of fully connected layers. Finally, the probability of lMCI and the hazard rate of AD conversion are calculated simultaneously as multitask learning. The main contributions of our studies are as follows:

- We proposed a multimodal multitask learning based approach to synchronously classify eMCI and lMCI stages in AD patients and predict the time to AD conversion from these MCI patients for early diagnosis of AD. To the best of our knowledge, this is the first study to integrate two tasks: the categorization of the MCI stage and the prediction of the period from the MCI stage to the onset of AD.
- Technically, we proposed a novel attention-based mechanism, SPAN, to learn data representations from finite sample datasets in a practical and effective manner.
- We carried out analysis of the exploratory investigation of radiomics characteristics for predicting the course of AD in three brain areas (GM, WM, and CSF).
- We proposed the use of a decay factor, AED, to aid in the acquisition of the dominant representation across modalities.
- We experimented on a public dataset and employed cross-validation to show the generalization of the proposed system. Several aspects of disease analysis were exploited to understand the course of AD better.

## Results

### Study participants

To evaluate the efficiency of the proposed framework, we used the Alzheimer’s Disease Neuroimaging Initiative (ADNI) cohort, which includes diagnosis of 1, 737 patients (ages 54.5 to 98.6 years) from 2005 to 2017. According to our scopes, which focus on the tasks of MCI-stage classification and time-to-AD prediction, we only selected patients who are diagnosed as ether eMCI or lMCI at baseline timepoint. Furthermore, we cleaned up the raw data through removing timepoints that had been ether duplicated or had implausible measurements, and we screened out irreversible individuals who had altered their condition from AD to MCI or from MCI to cognitive normal (CN) during the course of the study’s history.

Bases on given patients’ IDs, we manually collected their corresponding MRI scans from the ADNI site. In total, we obtained 249 eMCI and 427 lMCI patients at baseline diagnosis. Table 1 presents the collected data statistic from the ADNI cohort for two groups of eMCI and lMCI patients. There are significant differences between the two groups in terms of age, Clinical Dementia Rating Scale-Sum of Boxes (CDRSB), Mini Mental State Examination (MMSE), Alzheimer’s Disease Assessment Scale-13 (ADAS13), Rey Auditory Verbal Learning Test (RAVLT), Functional Activities Questionnaire (FAQ), volumetric and PET biomarkers ($p<0.05$).

**Figure 1:**
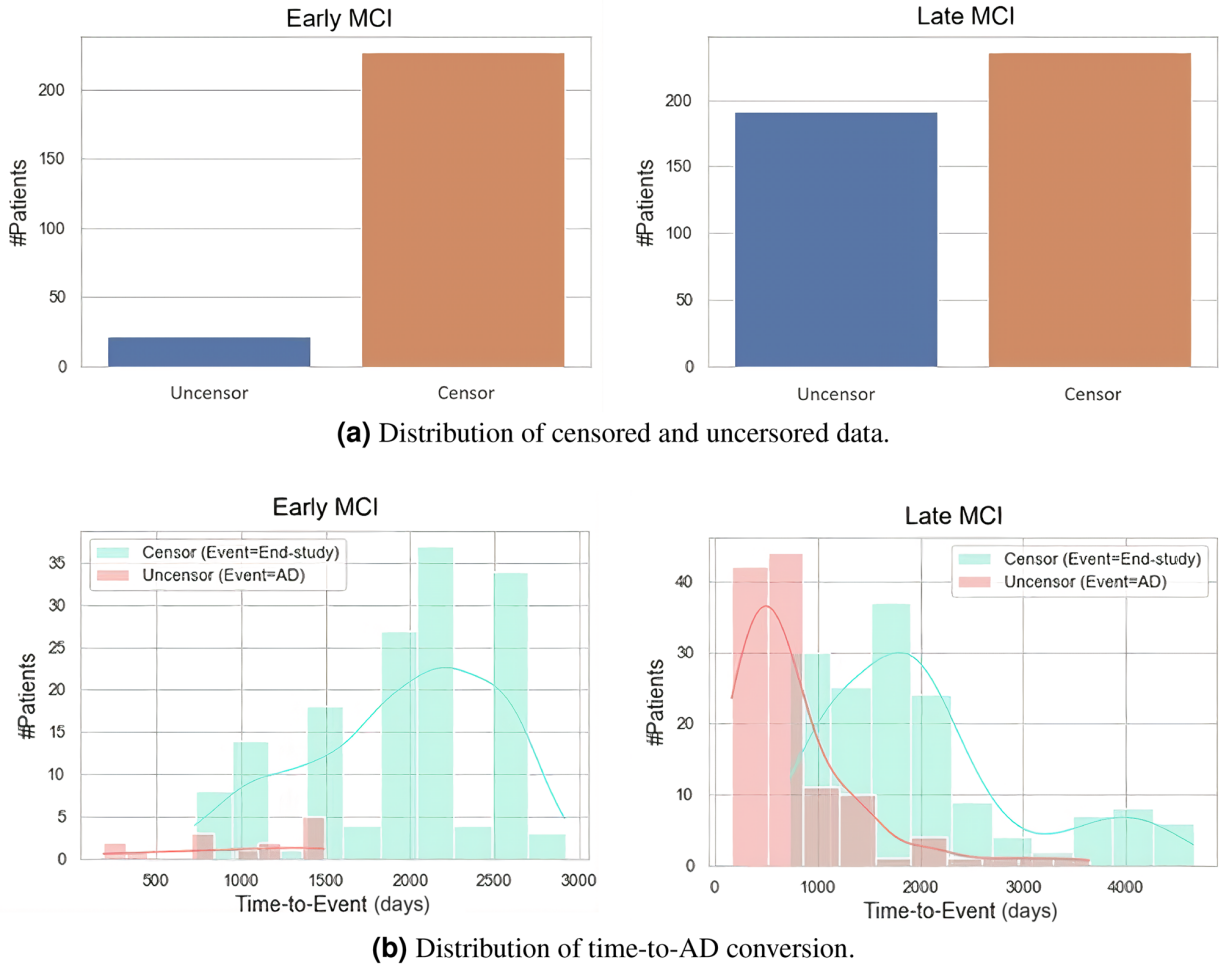
Data distribution.

To determine time-to-AD conversion, for uncensored patients, we assume that the conversion time is the time span between the baseline diagnosis and the first observation of AD. When considering the censored patients, the conversion time is calculated by adding the delaying time to their most recent visits. The data distribution and conversion time are visualized in Fig. 1. For the eMCI cases, the censored patients outnumber the uncensored ones. In Fig. 1b, the uncensored patient event occurs when the patient is diagnosed with AD, while the censored patient event occurs at the end of the study, which is the last observation on this patient. The details of data distributions are presented in the *Supplementary Materials*, section *Data distribution*. In addition, the model settings can be found in the *Supplementary Materials*, section *Experimental settings*.

**Table 1:** Data statistic of the collected ADNI cohort in this study.

Variable	eMCI (n=249)	lMCI (n=427)	*p* value
Male, gender, n (%)	141 $\left( 56.6\% \right)$	262 $\left( 61.4\%\right)$	$0.259^{*}$
Age, years, mean±std	$71.8\pm 7.3$	$74.5\pm 7.3$	4.1e-$6^{\bot }$
Education, years, mean±std	$15.9\pm 2.7$	$15.9\pm 3$	$0.959^{\bot }$
CDRSB, n (%)			$0.003^{*}$
$\le 1$	136 $\left( 54.6\% \right)$	181 $\left( 42.4\% \right)$	
MMSE, mean±std	$28.2\pm 1.6$	$27.2\pm 1.8$	2.2e-$13^{\bot }$
ADAS13, mean±std	$13.0\pm 5.3$	$18.6\pm 6.7$	6.7e-$21^{\bot }$
RAVLT, mean±std	$5.2\pm 2.4$	$3.5\pm 2.4$	2.5e-$19^{\bot }$
FAQ, mean±std	$2.0\pm 2.9$	$3.7\pm 4.5$	$0.001^{\bot }$
Volumetric biomarker, ml, median (IQR)			
Ventricles, $\times 10^3$	31.2 $\left[ 19.6, 46.9 \right]$	39.1 $\left[ 27.1, 57.7\right]$	9.0e-$6^{\Finv }$
Hippocampus, $\times 10^3$	7.2 $\left[ 6.6, 7.8\right]$	6.5 $\left[ 5.8, 7.2\right]$	4.6e-$5^{\Finv }$
Entorhinal, $\times 10^3$	3.8 $\left[ 3.3, 4.2\right]$	3.4 $\left[ 2.8, 3.9\right]$	4.5e-$4^{\Finv }$
Fusiform, $\times 10^3$	18.7 $\left[ 16.8, 20.4\right]$	16.9 $\left[ 15.0, 18.4\right]$	4.0e-$7^{\Finv }$
Middle Temporal, $\times 10^3$	20.5 $\left[ 19.1, 22.5\right]$	19.0 $\left[ 17.1, 21.0\right]$	5.5e-$5^{\Finv }$
Whole Brain, $\times 10^5$	10.6 $\left[ 9.9, 11.3\right]$	10.0 $\left[ 9.3, 10.9\right]$	1.3e-$8^{\Finv }$
PET biomarker, median (IQR)			
FDG	1.3 $\left[ 1.21, 1.35\right]$	1.2 $\left[ 1.13, 1.3\right]$	2.5e-$3^{\Finv }$
AV45	1.1 $\left[ 1.01, 1.31\right]$	1.3 $\left[ 1.07, 1.5\right]$	$0.^{\Finv }$

IQR: Interquartile Range.

$^{*}$ Chi-square test.

$$^{\bot }$$ T-test.

$^{\Finv }$ Mann-Whitney U test

For quantitative evaluation, we use c-index score (CI), Brier score (BS), and mean absolute error (MAE) as criteria of time prediction task, while using accuracy (Acc), average precision (AP), precision (Pre), recall (Rec), F1-score ($\hbox {F}_1$), and area under receiver operating characteristic curve (AUC) as criteria of classification task. For generalization, experiments are performed by 5-fold cross validation. The details of evaluation metrics are expressed in the *Supplementary Materials*, section *Evaluation metrics*.

### Comparison to conventional studies

In this section, we present a comprehensive analysis of the performance of our proposed model and the existing research in terms of prediction of time-to-AD conversion and MCI-stage classification, as shown in Tables 2 and 3. Table 2 highlights the performance of various methods in prediction time-to-AD conversion, including our proposed approach, along with the modalities, data size, and evaluation metrics employed. The study by Polsterl et al.^35^ utilized 3D hippocampus data along with clinical data to predict the conversion time to AD. Their approach achieved a CI of 0.803. Lu et al.^36^ focused on MRI and genetic data as their modalities for conversion time prediction. They reported a CI of 0.681, indicating moderate predictive performance. The BS, a measure of calibration, was reported as 0.147, suggesting some room for improvement in calibration. Nakagawa et al.^37^ employed gray matter (GM), patient age, and Mini-Mental State Examination (MMSE) scores as input features for their prediction model. Their approach achieved a CI of 0.83 when evaluating on both NC and MCI patients, and a CI of 0.75 when using MCI set only. Ho et al.^38^ investigated the use of demographics and brain biomarkers for conversion time prediction. Their approach achieved a CI of 0.804, similar to the performance reported by^35^. The BS value for this study was reported as 0.153, indicating good calibration. In comparison, our proposed method utilized radiomics features extracted from MRI scans along with clinical data for conversion time prediction of MCI patients. Our approach achieved a significantly higher CI of 0.846, indicating improved predictive performance compared to the other methods discussed. Additionally, the BS value for our approach was reported as 0.132, suggesting good calibration and accurate estimation of conversion time.

Table 3 summarizes the Acc and AUC values achieved by various methods for the classification of eMCI and late lMCI. Suk et al.^39^ utilized MRI and PET modalities for the classification of eMCI and lMCI, achieving an accuracy of $75.92\%$ and an AUC of 0.75. This approach performed reasonably well in distinguishing between the two classes, although the AUC suggests room for improvement in capturing the discriminatory power of the model. Nozadi et al.^40^ also employed MRI and PET data for classification, with a dataset of 164 eMCI and 189 lMCI samples. Their approach achieved an accuracy of $65.2\%$, indicating moderate performance in distinguishing between the two classes. Jie et al.^41^ focused on resting-state functional MRI (rs-fMRI) as their modality for classification. Their approach demonstrated a higher accuracy of $78.8\%$ and an AUC of 0.78. These results indicate better discrimination between eMCI and lMCI compared to the previous approaches utilizing MRI and PET. Zhang et al.^42^ also utilized rs-fMRI data for classification and achieved an accuracy of $83.87\%$ and an impressive AUC of 0.9, demonstrating superior performance in accurately distinguishing between the two classes. Nevertheless, their study was performed using an extremely limited dataset comprising only 33 eMCI and 29 lMCI patients, which makes it susceptible to issues of overfitting and lack of generalizability. Mehmood et al.^18^ focused on gray matter (GM) features derived from MRI scans for classification. Using a small dataset and fixing classes to be balanced with 70 eMCI and 70 lMCI samples, their approach achieved an accuracy of $83.7\%$. By training model on 2D images, they presented slower training and inference time compared to using input features as vector. Cui et al.^43^ achieved an accuracy of $76.13\%$ when evaluating with a small dataset of 45 eMCI and 5 lMCI patients. In comparison, our proposed method leveraged radiomics features extracted from MRI scans along with clinical data for eMCI and lMCI classification. Our approach achieved an accuracy of $83.19\%$ and an AUC of 0.91, demonstrating robust performance in accurately distinguishing between the two classes. The large dataset size of 249 eMCI and 427 lMCI samples further enhances the reliability of our results. In general, the experimental results indicate that our proposed method demonstrates promising approach for multitask learning of predicting time-to-AD conversion and classifying MCI-stage.

**Table 2:** Performance for conversion-time-to-AD prediction in terms of CI and BS; where $\uparrow$ : higher is better; $\downarrow$ : lower is better. The boldface values indicate the best performance.

Approach	Modality	Dataset	Prediction criteria	
			CI$\uparrow$	BS$\downarrow$
Polsterl et al.^35^	3D Hippocampus Clinical	397 MCI patients - Censoring rate: unknown	0.803	−
Lu et al.^36^	MRI Genetic	173 MCI patients - Censoring rate: $89\%$	0.681	0.147
Nakagawa et al.^37^	GM (MRI) Age, MMSE	2, 142 patients (NC, MCI) - Censoring rate: $75\%$ 1, 211 MCI patients - Censoring rate: $58.6\%$	0.83 0.75	−
Ho et al.^38^	Demographics Biomarkers	1, 334 patients (NC, MCI, AD) - Censoring rate: $74\%$	0.804	0.153
Ours (SPAN-AED)	Radiomics (MRI) Clinical	249 eMCI patients - Censoring rate: $92\%$ 427 lMCI patients - Censoring rate: $55.3\%$	$\mathbf {0.846}$	$\mathbf {0.132}$

**Table 3:** Performance for early MCI and late MCI classification in term of Acc and AUC; where $\uparrow$ : higher is better. The boldface values indicate the best performance.

Approach	Modality	Dataset	Classification criteria	
		(eMCI / lMCI)	Acc$\uparrow$	AUC$\uparrow$
Suk et al.^39^	MRI & PET	128 / 76	75.92	0.75
Nozadi et al.^40^	MRI & PET	164 / 189	65.2	−
Jie et al.^41^	rs-fMRI	56 / 43	78.8	0.78
Zhang et al.^42^	rs-fMRI	33 / 29	$\mathbf {83.87}$	0.9
Mehmood et al.^18^	GM (MRI)	70 / 70	83.7	−
Cui et al.^43^	MRI	45 / 51	76.13	−
Ours (SPAN-AED)	Radiomics (MRI) Clinical	249 / 427	83.19	$\mathbf {0.91}$

### Performance on prediction of MCI to AD conversion

We individually investigated clinical and radiomics characteristics to determine the effectiveness of our proposed model compared to the unimodal approach. In the *Performance on combination of radiomics features* subsection of the *Supplementary Materials*, we demonstrated that the optimal combination of $\left[ CSF \right] \left[ texture \right]$ features was utilized as the radiomics input for our proposed model. In comparison to the study conducted by Ho et al.^38^, we replaced the SPAN module with a residual-attention (RA) module for feature encoding. Our SPAN algorithm exhibited superior performance in predicting conversion time-to-AD with clinical features, achieving a higher CI (0.82 compared to 0.8) and lower MAE (454 days compared to 510 days). Additionally, it slightly improved the performance of the MCI-stage classification task. Notably, the SPAN encoder outperformed the RA encoder in both tasks, resulting in a reduction of 56 days in MAE and an improvement of $0.89\%$ in accuracy. Instead of the proposed AED fusion strategy, we utilized concatenation (Concat) for comparison. Experimental results showed that our proposed AED approach outperformed traditional concatenation in representation fusion for both tasks. It yielded a 0.02 increase in CI (using SPAN encoder), a reduction of up to 59 days in MAE (using RA encoder), a reduction of up to 8 days in MAE (using SPAN encoder), and an increase of up to $0.67\%$ in accuracy (using RA encoder and SPAN encoder). The detailed results of the prediction and classification tasks for MCI to AD conversion are presented in Table 4. In conclusion, multimodal approaches surpassed the use of unimodal approaches, both for clinical and radiomics features. Moreover, the SPAN module consistently outperformed the RA module. Integrating SPAN with AED further enhanced performance compared to utilizing SPAN with the Concat operation.

In addition, further analysis of performance on combination of radiomics features and visualization of time-to-AD conversion are described in the *Supplementary Materials*, section *Ablation studies*.

**Table 4:** Performance for conversion time-to-AD prediction and eMCI vs lMCI classification for unimodal and multimodal approaches; where $\uparrow$ : higher is better; $\downarrow$ : lower is better. The boldface values indicate the best performance.

Modality	Approach	Prediction Criteria			Classification criteria					
		CI$\uparrow$	BS$\downarrow$	MAE$\downarrow$	Acc$\uparrow$	AP$\uparrow$	Pre$\uparrow$	Rec$\uparrow$	$\hbox {F}_1\uparrow$	AUC$\uparrow$
Clinical	RA	0.8	0.16	510	80.83	91.22	80.67	80.54	80.24	0.88
	SPAN	0.82	0.16	454	81.16	91.53	81.32	81.77	81.53	0.88
Radiomics	RA	0.67	0.2	725	77.21	92.37	76.47	78.23	77.54	84.42
	SPAN	0.67	0.19	669	78.1	92.64	77.81	78.65	78.13	85.46
Multimodal	RA-Concat	0.82	0.16	445	82.35	94.63	80.94	81.86	81.32	0.89
	RA-AED	0.82	0.14	386	83.02	93.68	82.25	${\textbf {83.44}}$	${\textbf {82.51}}$	0.89
	SPAN-Concat	0.83	0.13	355	82.89	94.14	${\textbf {83.23}}$	82.36	82.23	0.9
	Ours(SPAN-AED)	${\textbf {0.85}}$	${\textbf {0.13}}$	${\textbf {347}}$	${\textbf {83.19}}$	${\textbf {94.76}}$	82.31	82.85	82.47	${\textbf {0.91}}$

## Discussion

Recent neuroimaging studies revealed that individuals diagnosed with MCI and AD have considerable disruption in either the structural network or the functional network when compared to a healthy control group^17,44^. Few studies have investigated the features of whole brain networks in patients with MCI at various stages of the disease. Zhang et al.^42^ utilized the graph theory to measure the relationship between changes in the brain network connectivity from the resting-state fMRI. Then, the support vector machine (SVM) was used to distinguish eMCI from lMCI at different frequency bands, and achieved the best performance in slow-5 band with a $83.87\%$ accuracy. Transfer learning approaches are usually used to overcome privacy and cost issues for a massive quantity of annotated data, which entails applying a pre-trained model to new problems using a smaller dataset. By taking the advantage of these facts, Mehmood et al.^18^ developed a layer-wise transfer learning model based on VGG architecture family^45^ to segregates between eMCI and lMCI and achieved a $83.72\%$ accuracy. Cui et al.^43^ proposed two-stage algorithm based on particle swarm optimization (PSO) for removing redundant features and adaptive LASSO logistic regression model for selecting the most relevant features to predict AD stages.Experimental results have been shown a $76.13\%$ accuracy on stable MCI (sMCI) vs converted MCI (cMCI) patients.

A survival analysis is a type of statistical study that examines time-to-event data, which describes the period between a time origin and an endpoint of particular interest^46^. Polsterl et al^35^ proposed a wide and deep neural network for survival analysis that learns to detect individuals who are at a high risk of advancing to AD using information from 3D hippocampal geometry and tabular clinical data. According to their findings, tabular clinical makers with a median c-index of 0.750 are already good predictors of conversion from MCI to AD. In addition, in the hippocampus volume, the median c-index climbed to 0.803 when the hippocampus volume was included. Nakagawa et al.^37^ discovered a deep learning method-based survival analysis could be used to assess the likelihood that an individual will get AD over a particular period of time. They approached the survival problem in a unique way and demonstrated encouraging results across many cohorts. Ho et. al^38^ proposed a modification of DeepSurv architecture^47^, called RASurv to analyze the time-to-AD conversion for both cognitive normal and MCI patients. Their model achieved a competitive performance to other methods with a c-index score of 0.804.

The difficulty of precisely determining when an individual transitioned to AD can be attributed to the no studies on the topic of prediction of time-to-AD conversion. Typically, the occurrence happens before an individual is diagnosed as AD. However, we usually assume that the event occurs at the timepoint that the patient is diagnosed as conversion from MCI to AD to alleviate the problem. The above-conventional study focused exclusively on single tasks, despite the possibility of a correlation between MCI phases and time-to-AD conversion. In general, an eMCI patient has a lower risk of developing AD within a short period of time than an lMCI patient. As a result, it is essential to master two tasks concurrently: MCI-stage classification and conversion-time-to-AD prediction. In addition, the criteria for eMCI and lMCI can be found in the *Supplementary Materials*, section *Criteria for the MCI stages*.

This study presented a novel framework of multimodal multitask learning to discriminate eMCI patients from lMCI patients and forecast conversion time till the onset of Alzheimer’s disease. The proposed model derived features from clinical representations (which include patient information, cognitive measurements, and biomarkers) as well as radiomics representations (which are estimated from brain MRI). The SPM program was used to normalize brain MRI dimension and segment three different brain regions: the GM, WM, and CSF, in particular. These regions’ masks combined with brain image were used to determine the shape and texture of radiomics characteristics with the PyRadiomics program. We proposed SPAN (stacked polynomial attention network) to effectively and reliably capture the approximation basis for all polynomials of constrained degree. The clinical and radiomics characteristics were supplied into two branches of SPAN and dropout series, which were then used to encode the appropriate information in the patient’s medical record. After that, we constructed an adaptive exponential decay (AED) factor to combine the encoded representations from both branches together. We evaluated the proposed model on the ADNI cohort that overcomes the state-of-the-art performance.

However, it is essential to obtain radiomics characteristics from MRI scans to lower the dimension of the 3D images due to the large batch size required for ranking optimization in this study. However, the performance of radiomics characteristics is significantly low when compared to clinical data as shown in Table 1. This means that radiomics features may contribute less to the multi-model and may potentially introduce bias into the overall network. As a result, in future studies, we will examine other strategies for extracting more robust representations of 3D images. Furthermore, we analyzed global brain regions (such as the GM, WM, and CSF) in this study, despite the fact that there are critical areas (such as frontal lobe, motor cortex, sensory cortex, parietal lobe, occipital lobe, and temporal lobe, etc.) that often influence AD conversion. Therefore, future studies will include examining the relationships between brain areas.

Overall, we firmly believe that our study holds important value in the field of AD diagnosis and understanding. Our proposed multimodal multitask learning approach, attention-based mechanism, and exploratory investigation of radiomics characteristics provide valuable insights and potential avenues for early diagnosis and improved understanding of the course of AD. By integrating tasks, improving data representations, and incorporating multimodal information, we aim to advance the field’s understanding of AD progression and contribute to the development of more effective diagnostic tools.

## Methods

**Figure 2:**
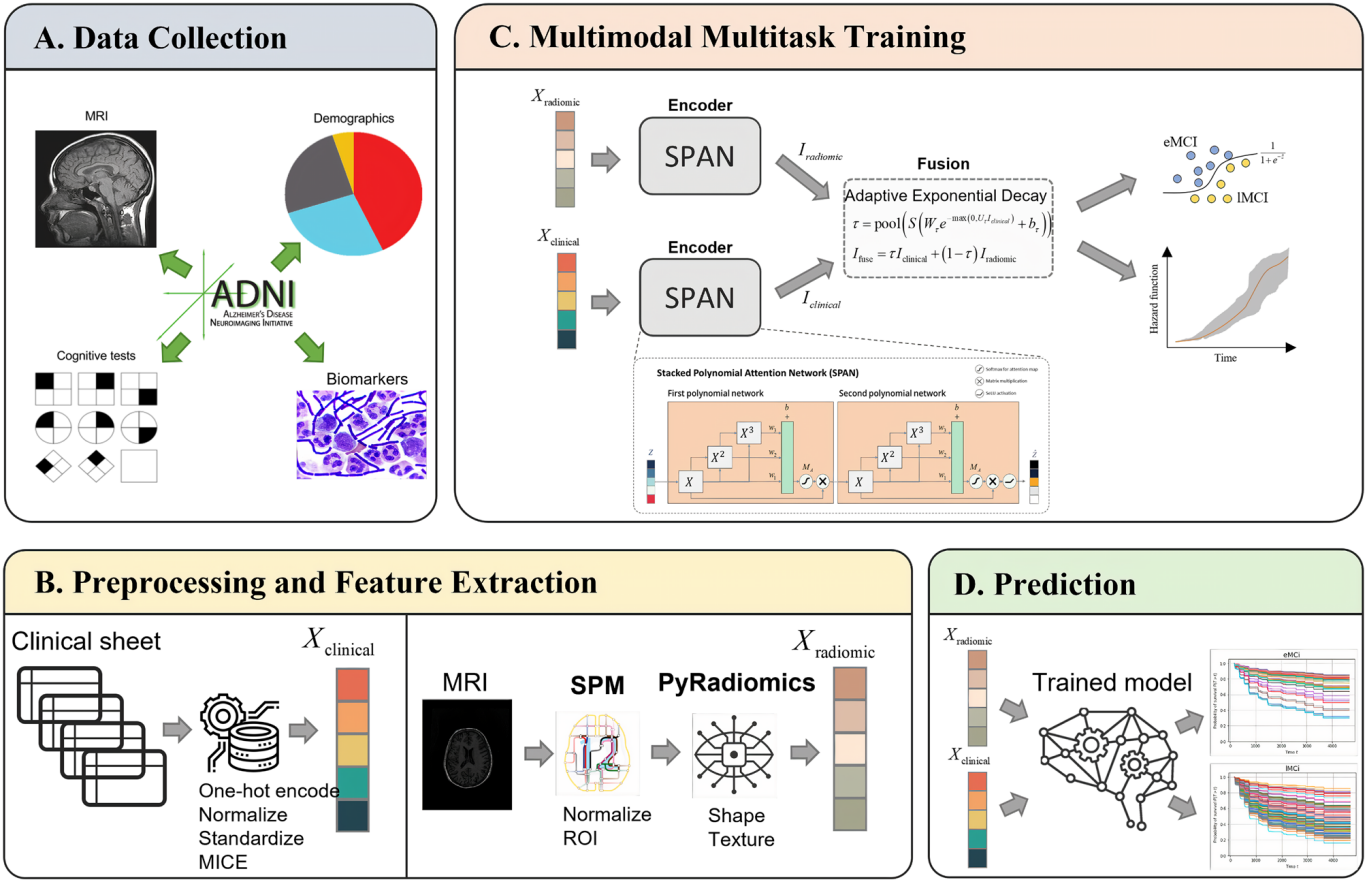
Overall process of the proposed method for MCI-stage classification and conversion-time-to-AD prediction. (**A**) Collecting data from ADNI cohort, (**B**) preprocessing clinical data and extracting radiomics features from MRI images, (**C**) training multimodal multitask model with the proposed SPAN network and AED fusion module, and (**D**) predicting time-to-AD conversion for both eMCI and lMCI patients.

We developed a new paradigm for identifying MCI stages and predicting time-to-AD conversion using clinical and radiomics characteristics. First, we preprocess the raw clinical data and estimate radiomics features from MRI scans. Then, we encode the clinical and radiomics representations using a succession of SPAN and dropout layers. The AED algorithm efficiently fuses the two branches’ prominent characteristics predict the probability of eMCI vs lMCI phases and the hazard rate of AD conversion. The overall process is shown in Fig. 2. *Note that this article does not contain any studies involving animals or human participants performed by any of the authors*.

### Preprocessing and feature extraction

Numerous studies on Alzheimer’s disease dementia make use of information obtained through expensive and invasive techniques such as brain imaging or spinal taps to predict the risk of getting Alzheimer’s disease dementia or fast cognitive decline in the future. A low-cost and noninvasive approach to studying the evolution of Alzheimer’s disease dementia might be based on clinical data (e.g., demographics, vital signs, medicines, laboratory data, vital signs, and current medical problems). Because clinical data can be supplied in a variety of forms, it is necessary to do data preprocessing and transformation prior to training a model on clinical data. In this study, we perform one-hot encoding for transforming categorical data and z-score normalization for infinitive numerical values and maximum normalization for limited numerical values, excluding volumetric biomarkers, which are scaled by dividing the total intracranial volume (ICV) of each individual. Since clinical data commonly appear with missing data in medical studies when the value of the variables of interest is not measured or recorded for all of the participants in the sample, we utilize the Multiple Imputation by Chained Equations (MICE) algorithm^48^ to impute the missing values.

Radiomics, which is based on the high-dimensional quantification of medical scans and enables the retrieval of more precise features than standard visual interpretation, can reveal information for treatment interventions^49,50^. There has been some investigation into the use of radiomics in identifying the progression of AD^51,52^. These investigations revealed that radiomics biomarkers can be used to classify individuals with MCI who are at high risk of developing Alzheimer’s disease in the future. Furthermore, radiomics biomarkers in combination with clinical analysis can vastly enhance the prediction accuracy of MCI to AD. To extract the radiomics features, we first utilize the “Normalization” module of SPM toolbox to scale the intensity and space of the three-dimension MRI image since the brain structure varies from person to person. Next, we segment the normalized brain into three regions such as GM, WM, and CSF using the “Segmentation” module. Then, we extract the various types of radiomics features, which can be divided into shape and texture groups, using the PyRadiomics tool.

In our approach, we first normalize and standardize all features to ensure that features from different scales or with varying distributions were placed on a comparable scale, that prevent any single feature from dominating the learning process and promote fair contributions from all features. Next, we concatenate all features of each type of representation, namely radiomics and clinical, to create a single feature vector for each representation. This concatenation step ensures that all relevant information from the respective feature sets is preserved. The details of clinical data preprocessing and radiomics extraction can be found in the *Supplementary Materials*, section *Clinical and radiomics features preprocessing*.

### Stacked polynomial attention network (SPAN)

Recent years have seen an increase in the use of attention mechanisms to not only improve the performance but also the explainability of deep learning techniques. Initially, the attention mechanism was mostly employed to describe sequence dependencies independent of their real distances^53,54^. Abd Hamid et al.^55^ used an attention mechanism and a global average pooling (GAP) layer to extract the most prominent information from an MRI image for the purpose of differentiating between AD states. In previous study^56^, researchers stated that the stacked deep polynomial network (S-DPN) can enhance the representation performance of the retrieved characteristics and held promise for the neuroimaging-based AD diagnosis. Based on these findings, we developed a novel attention mechanism based on S-DPN and dubbed the stacked polynomial attention network (SPAN) for exploiting attended representation from constrained indeterminates. Given an input feature *Z*, the sequential expressions of the first polynomial network of the SPAN module are as follows:

1 $$\begin{aligned} {} & X_{1}^{(1)}=W_{Z}^{(1)}Z+b_{Z}^{(1)} \\&X_{2}^{\left( 1 \right) }=X_{1}^{(1)}\times X_{1}^{(1)} \\&\cdots \\&X_{n}^{(1)}=X_{n-1}^{(1)}\times X_{1}^{(1)} \\&M_{A}^{(1)}=\sigma \left( w_{n}^{(1)}X_{n}^{(1)}+\ldots +w_{1}^{(1)}X_{1}^{(1)}+{{b}^{(1)}} \right) \\&{{{\hat{Z}}}^{(1)}}=X_{1}^{(1)}\times M_{A}^{(1)} \\ \end{aligned} $$

where $X_{1}^{(1)}, X_{2}^{(1)},\ldots , X_{n}^{(1)}$ represent indeterminates of polynomial function with *n* degrees of the first network, $W_{Z}^{(1)}, b_{Z}^{(1)}, w_{1}^{(1)},\ldots ,w_{n}^{(1)}, {{b}^{(1)}}$ represent trainable parameters, $\sigma \left( \right)$ is softmax function for generating attention map $M_{A}^{(1)}$, and ${{{\hat{Z}}}^{(1)}}$ represents the attended representation. ${{{\hat{Z}}}^{(1)}}$ is then fed to the second polynomial network, which is similar to the first one, to stack up feature representation and yield a better and deeper structure. The scaled exponential linear unit (SELU) activation function (^57^) is then used to add non-linearity to the neural network. The sequence of the second polynomial network is expressed as follows:

2 $$ \begin{aligned}{} & X_{1}^{(2)}=W_{Z}^{(2)}{{{\hat{Z}}}^{(1)}}+b_{Z}^{(2)} \\&X_{2}^{\left( 2 \right) }=X_{1}^{(2)}\times X_{1}^{(2)} \\&\cdots \\&X_{n}^{(2)}=X_{n-1}^{(2)}\times X_{1}^{(2)} \\&M_{A}^{(2)}=\sigma \left( w_{n}^{(2)}X_{n}^{(2)}+\ldots +w_{1}^{(2)}X_{1}^{(2)}+{{b}^{(2)}} \right) \\&{{{\hat{Z}}}^{(2)}}=\text {SELU}\left( X_{1}^{(2)}\times M_{A}^{(2)} \right) \\ \end{aligned}$$

### Multimodal fusion network

**Figure 3:**
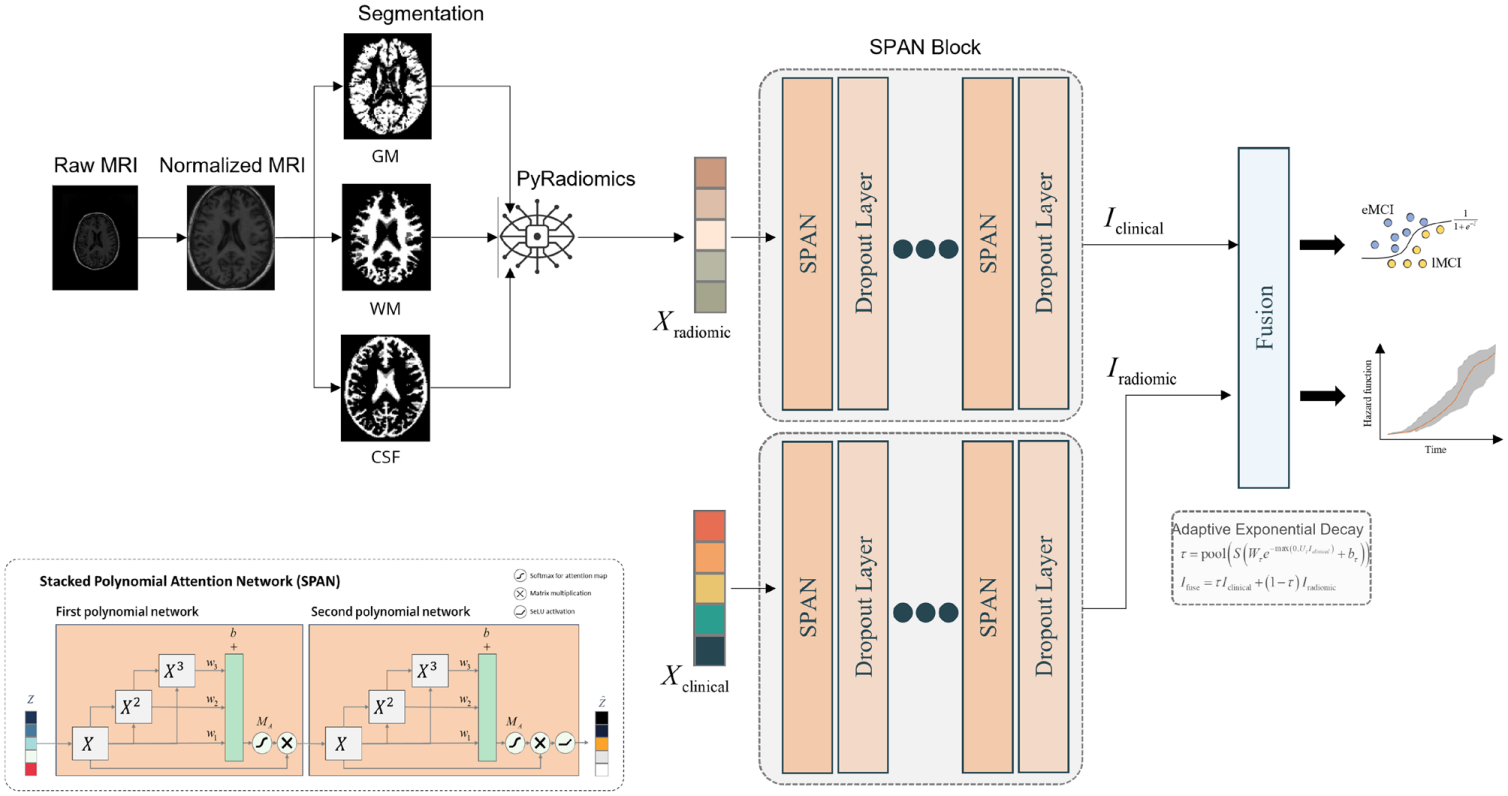
Architecture of the proposed multimodal multitask learning with SPAN.

Multimodal data can help improve the accuracy of diagnosis, prediction, and overall performance of learning systems (^58^). For instance, Venugopalan et al.^59^ proposed multimodal deep learning models for AD data fusion to improve AD stage identification. Their trials established that the multimodal strategy outperformed the unimodal approach. De Jesus Junior et al.^60^ described the discovery of multimodal indicators of AD severity for individuals in the early stages of the disease through combining Resting-State EEG and structural MRI data. In addition, their findings demonstrated the efficacy of the multomodal strategy. In this study, we present the multimodal multitask architecture for classifying MCI stage and predicting time-to-AD conversion. Our proposed model has two branches, as shown in the Fig. 3. The first branch operates on radiomics features, which are generated from a 3D MRI image using the SPM and PyRadiomics toolboxes, while the second branch operates on preprocessed clinical data. Each branch is connected to a SPAN block, which is comprised of a series of SPAN-followed dropout layers.

Assume that $I_\text {radiomic}$ and $I_\text {clinical}$ are the output features from SPAN blocks of radiomics and clinical features, respectively, we define an adaptive factor to select superior candidates from both representations. The operations for multimodal fusion are expressed as follows:

3 $$ \begin{aligned}{} & \tau =\text {pool}\left( S \left( {{W}_{\tau }}{{e}^{-\max \left( 0, {{U}_{\tau }}{{I}_{\text {clinical}}} \right) }}+{{b}_{\tau }} \right) \right) \\&{{I}_{\text {fused}}}=\tau {{I}_{\text {clinical}}}+\left( 1-\tau \right) {{I}_{\text {radiomic}}} \\ \end{aligned} $$

where $\tau$ represents adaptive exponential decay (AED), ${{W}_{\tau }}, {{U}_{\tau }}, {{b}_{\tau }}$ represent trainable parameters, $\text {pool}\left( \cdot \right)$ represents maximum pooling operation, $S\left( \cdot \right)$ represents sigmoid function, and ${{I}_{\text {fused}}}$ represents the fused representation from clinical and radiomics features. We used the exponential negative rectifier for decay factor $\tau$ to ensure that each decay rate decreases asymptotic within a tolerable range of 0 to 1. Lastly, the fused features is used to immediately predict the probabilities of eMCI vs lMCI, $\hat{y}_\text {pr}$, and the hazard rate of AD conversion, $\hat{y}_\text {hr}$, as follows:

4 $$\begin{gathered}   \hat{y}_{{pr}}  = S(W_{{cl}} I_{{fused}}  + b_{{cl}} ) \hfill \\   \hat{y}_{{hr}}  = W_{{hr}} I_{{fused}}  + b_{{hr}}  \hfill \\  \end{gathered}$$

where ${{W}_{cl}}, {{b}_{cl}}, {{W}_{hr}}, {{b}_{hr}}$ represent trainable parameters.

### Objective functions

To optimize the model’s cost, we joint two objective functions of two tasks: MCI-stage classification and conversion-time-to-AD prediction. For classification task of eMCI vs lMCI, each predicted probability to the actual class output is measured by the binary cross-entropy (BCE) (^61^). Once the score has been calculated, probabilities are penalized based on the distance from the predicted value. That indicates how near or far the actual number is from the estimate. Given the actual class $y_\text {pr}$ (0 for eMCI and 1 for lMCI), the BCE formula is as follows:

5 $$\begin{aligned} {\mathfrak {L}_{BCE}}=-\frac{1}{M}\sum \limits _{i=1}^{M}{\left( {{y}_{\text {pr}}}\centerdot \log \left( {{{\hat{y}}}_{\text {pr}}} \right) +\left( 1-{{y}_{\text {pr}}} \right) \centerdot \log \left( 1-{{{\hat{y}}}_{\text {pr}}} \right) \right) } \end{aligned}$$

where *M* is the number of samples within an iteration. Besides, we use the negative log-likelihood function^62^ to minimize model’s loss for the conversion-time-to-AD prediction task. Its expression is as follows:

6 $$\begin{aligned} {\mathfrak {L}_{NLL}}=-\frac{1}{{{M}_{E=1}}}\sum \limits _{i:{{E}_{i}}=1}{\left( {{{\hat{y}}}_{hr}}\left( i \right) -\log \left( \sum \limits _{j\in \mathbb {R}\left( {{T}_{j}} \right) }{{{e}^{{{{\hat{y}}}_{hr}}\left( j \right) }}} \right) \right) } \end{aligned}$$

In order to optimize the $\mathfrak {L}_{NLL}$, we need to maximize the term of ${\left( {{{\hat{y}}}_{hr}}\left( i \right) -\log \left( \sum \nolimits _{j\in \mathbb {R}\left( {{T}_{j}} \right) }{{{e}^{{{{\hat{y}}}_{hr}}\left( j \right) }}} \right) \right) }$ for each patient *i* having event $E=1$ (uncensored patient who is converted to AD) for every censored patient (non-converted to AD). It follows that we must raise the risk factor for every uncensored patient *i* while simultaneously lowering the risk factor for patients *j* who have not experienced the event until time $T_i$, which is the observed time-to-AD for patient *i*. Finally, we add both loss functions for simultaneously multitask learning and arrive at the following result:

7 $$\begin{aligned} {\mathfrak {L}_{total}} = {\mathfrak {L}_{BCE}} + {\mathfrak {L}_{NLL}} \end{aligned}$$

## Supplementary Information

Supplementary Information.

## Data Availability

The dataset generated and analysed during the current study are available in the *Test Data* section of the *Download | Study Data* section of the IDA website (https://ida.loni.usc.edu/pages/access/studyData.jsp?categoryId=43 &subCategoryId=94) under the name of *Tadpole challenge data*.
