# Supplementary material for: Multimodal multitask learning for predicting MCI to AD conversion using stacked polynomial attention network and adaptive exponential decay
Source: Sci Rep. 2023 Jul 11;13:11243. doi: 10.1038/s41598-023-37500-7 (PMC10336016; doi:10.1038/s41598-023-37500-7)
Supplement: Supplementary file 1 — Supplementary Information. [file 41598_2023_37500_MOESM1_ESM.pdf]

Multimodal Multitask Learning for Predicting MCI to AD Conversion using Stacked Polynomial Attention Network and Adaptive Exponential Decay: Supplementary Materials

DATA DISTRIBUTION

Fig. S1 illustrates the data statistics used in this study in terms of eMCI and lMCI groups. The two groups have comparable average ages and levels of education. Males outnumber females. Indeed, the lMCI group converted at a significantly greater rate than the eMCI group.

CRITERIA FOR THE MCI STAGES

According to ADNI studies (https://adni.loni.usc.edu/methods/documents/), there are six distinct criteria for identifying eMCI and lMCI, which are summarized in Table S1. As can be seen from this table, the majority of criteria are same between eMCI and lMCI, with the exception of the Wechsler Memory Scale–Revised score, which makes it extremely difficult to distinguish eMCI from lMCI.

CLINICAL AND RADIOMICS FEATURES PREPROCESSING

Fig. S2 displays the flow chart of radiomics extraction from MRI scans. First, we utilize the "Normalization" module of SPM to scale the intensity and space of the three-dimension MRI image since the brain structure varies from person to person. Next, we segment the normalized brain into three regions such as GM, WM, and CSF using the "Segmentation" module. Then, we extract the various types of radiomics features, which can be divided into shape and texture groups, using the PyRadiomics tool.

The clinical features with missing rate are shown in Table S2. There is no missing values for demographics, CDRSB, MMSE and RAVLT features.

The radiomics features extracting from the PyRadiomics are presented in Table S3. In general, there are 26-shape features and 75-texture features.

EXPERIMENTAL SETTINGS

All experiments were conducted using the NVIDIA RTX 3090 machine with 24GB memory. Each experiment was train on 300 epochs with the batch size of 128. We utilized the ADAM optimizer [1] for training deep learning models with modifications of learning rate and decay factor. All models were built under Python language and Tensorflow 2.7 framework. The details of hyper-parameters are introduced in Table S4.

Furthermore, to assess the generalization capability of our model, we employed a 5-fold cross-validation procedure, where we partitioned the available data into training, validation, and testing sets. Particularly, we divided the whole dataset into 5 folds, in which 1 fold is used as testing set and the remaining 4 folds are divided into training and validation sets with the ratio of 9 : 1. To address convergence issues, we monitored the model's training progress and employed early stopping based on the validation loss during 300 epochs. Then, we utilized the trained model with the best validation loss to predict for the testing set. This allowed us to estimate the model's performance on unseen data. Additionally, we employed L1_L2 regularization techniques to mitigate overfitting and improve generalization. This regularization technique combines L1 regularization, which encourages sparsity in the model by setting some feature weights to zero, and L2 regularization, which limits the magnitude of weight values. By incorporating this regularization approach, we aimed to encourage the network to focus on the most relevant features while reducing the likelihood of overfitting.

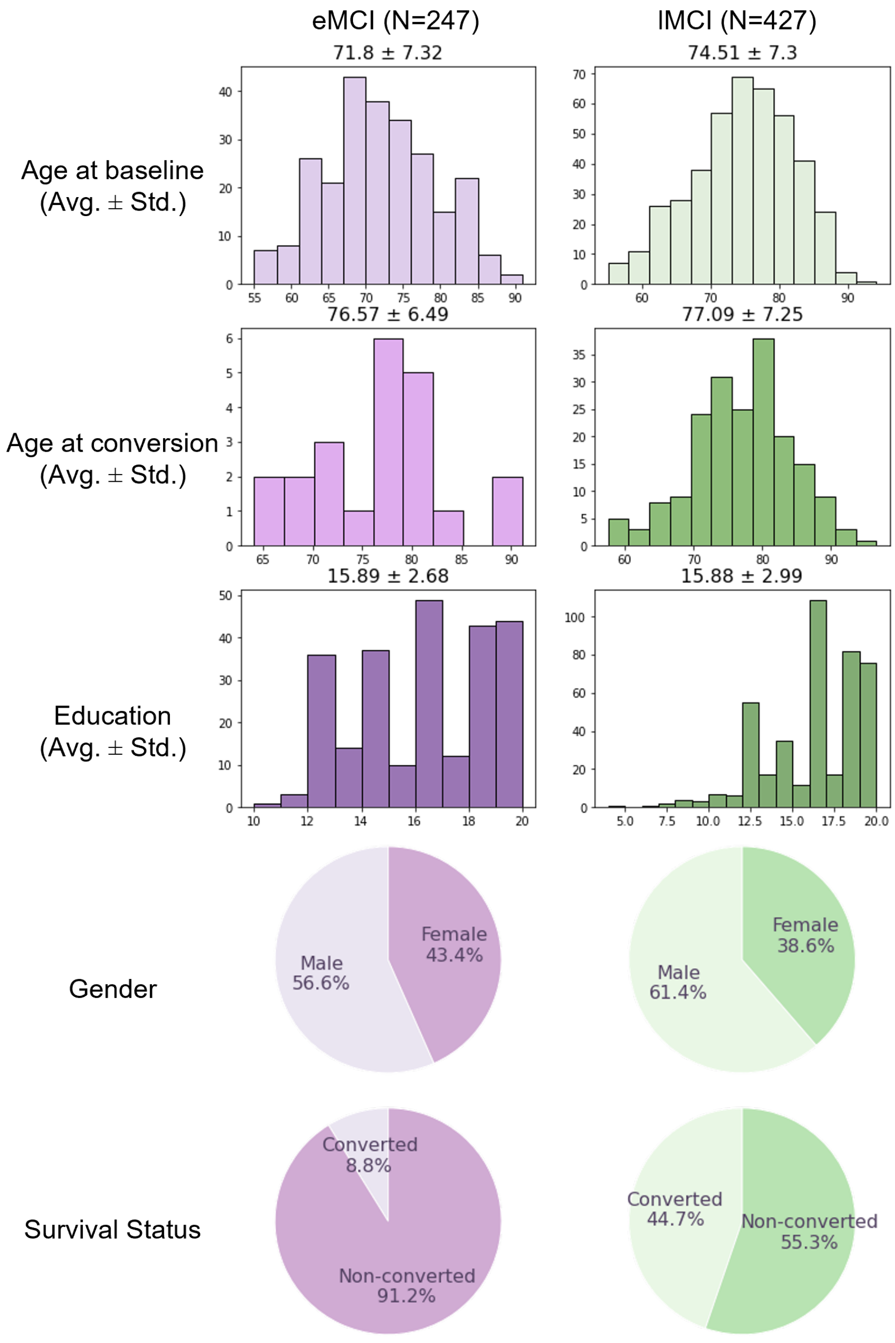

Fig. S1. Summary statistics of the patient in this study.

Table S1. Criteria for the MCI stages

Criteria Index	Early MCI	Late MCI
1	Must have a subjective memory concern as reported by subject, study partner, or clinician	Same as Early MCI
2	Wechsler Memory Scale-Revised:<br>• ≥ 16 years of education: $9-11$<br>• $8-15$ years of education: $5-9$<br>• $0-7$ years of education: $3-6$	Wechsler Memory Scale-Revised:<br>• ≥ 16 years of education: ≤ 8<br>• $8-15$ years of education: ≤ 4<br>• $0-7$ years of education: ≤ 2
3	Mini-Mental State Exam score: $24-30$	Same as Early MCI
4	Clinical Dementia Rating : 0.5<br>Memory Box score : 0.5	Same as Early MCI
5	General cognition and functional performance sufficiently preserved such that a diagnosis of Alzheimer's disease cannot be made by the site physician at the time of the screening visit	Same as Early MCI
6	Stability of Permitted Medications for 4 weeks:<br>• Antidepressants<br>• Estrogen<br>• Gingko Biloba<br>• Cholinesterase inhibitors and memantine	Same as Early MCI

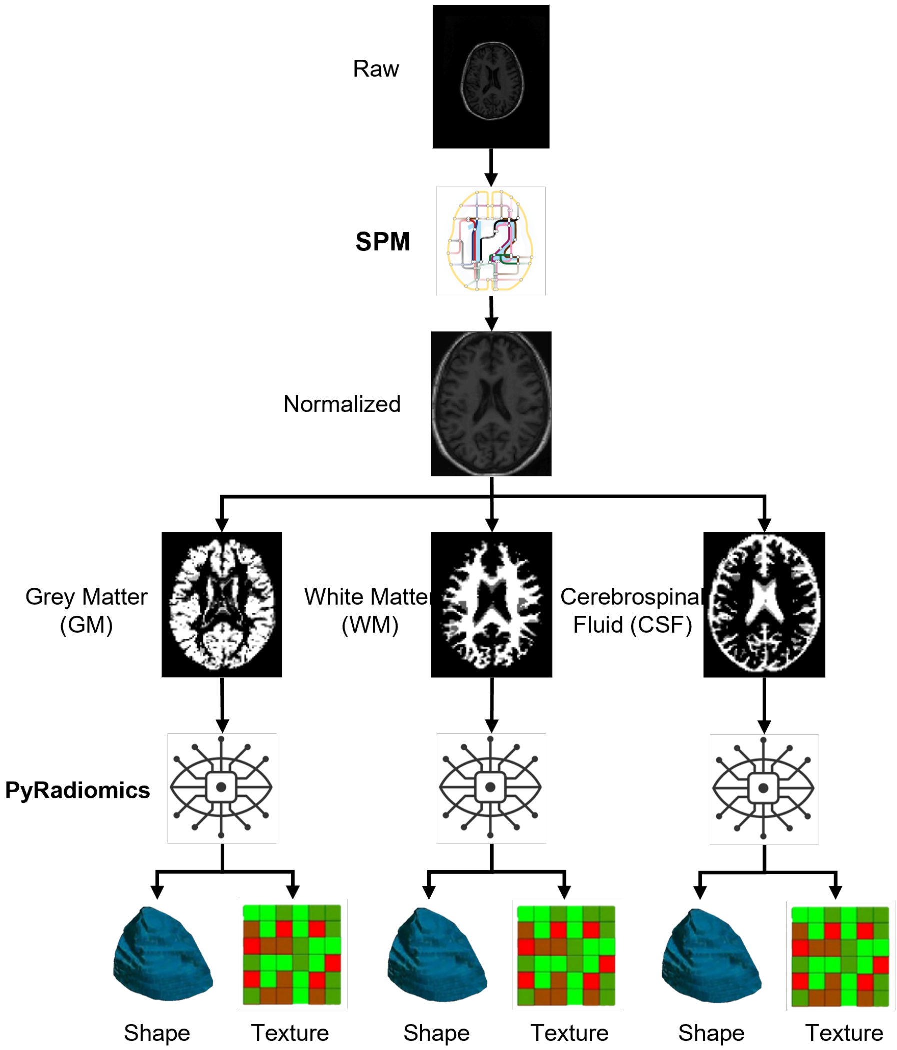

Fig. S2. Radiomics feature extraction from MRI scan using SPM and PyRadiomics.

Table S2. Preprocessing and missing rate of clinical data.

Feature	Type	Preprocessing	No. of preprocessed features	Missing rate (%)
Gender	Categoric	One-hot	1	0
Age	Numeric	z-norm	1	0
Education	Numeric	max-norm	1	0
Race	Categoric	One-hot	5	0
Ethnic	Categoric	One-hot	2	0
Marriage Status	Categoric	One-hot	4	0
Clinical Dementia Rating Scale–Sum of Boxes (CDRSB)	Numeric	max-norm	1	0
Alzheimer's Disease Assessment Scale–13 (ADAS13)	Numeric	max-norm	1	0.15
Mini Mental State Examination (MMSE)	Numeric	max-norm	1	0
Rey Auditory Verbal Learning Test (RAVLT)	Numeric	max-norm	1	0
Functional Activities Questionnaire (FAQ)	Numeric	max-norm	1	0.44
Ventricles	Numeric	ICV	1	4
Hippocampus	Numeric	ICV	1	15.38
Entorhinal	Numeric	ICV	1	15.82
Fusiform	Numeric	ICV	1	15.82
Middle Temporal	Numeric	ICV	1	15.82
Whole Brain	Numeric	ICV	1	2.07
Fluorodeoxyglucose (FDG)	Numeric	z-norm	1	22.34
Amyvid-45 (AV45)	Numeric	z-norm	1	45.56

One-hot: one-hot encoding for categorical data; z-norm: z-score normalization by mean and standard deviation; max-norm: dividing by maximum values; ICV: dividing by ICV value

EVALUATION METRICS

The accuracy (Acc) metric represents the overall effectiveness of a classifier and ban be expressed as follows:

$$\text{Acc} = \frac{TP + TN}{TP + FP + TN + FN} \tag{S1}$$

where TP is true positives, TN is true negatives, FP is false positives, and FN is false negatives.

The precision (Pre) metric stands for the class agreement between the data labels and the classifier's positive labels.

$$\text{Pre} = \frac{TP}{TP + FP} \tag{S2}$$

The recall (Rec) metric illustrates the ability of a classifier to recognize positive labels.

$$\text{Rec} = \frac{TP}{TP + TN} \tag{S3}$$

The F1-score (F_1) measures the harmonic meaning between precision and recall values.

$$\text{F}_1 = 2\frac{\text{Pre} * \text{Rec}}{\text{Pre} + \text{Rec}} \tag{S4}$$

The average precision (AP) determines The average of per class precision .

$$\text{AP} = \frac{\sum_{i=1}^{c} \frac{TP_i}{TP_i + FP_i}}{c} \tag{S5}$$

where c is the number of classes.

The area under the receiver operating characteristic curve (AUC) presents how well the probability ranks based on the true classes.

$$\text{AUC} = \frac{1}{(TP + FN) \cdot (FP + TN)} \sum_{i=1}^{TP+FN} \sum_{j=1}^{FP+TN} 1\left((+) - (-)\right) \tag{S6}$$

Table S3. Types of radiomics features.

Type	Group	No. of features
2D shape features	Shape	10
3D shape features	Shape	16
Gray level cooccurence matrix	Texture	24
Gray level run length matrix	Texture	16
Gray level size zone matrix	Texture	16
Neighbouring gray tone difference matrix	Texture	5
Gray level dependence matrix	Texture	14
	**Total**	101

Table S4. Model Hyper-parameters.

Variable		Clinical	Radiomics	Multimodal
#Input features		27	$26 \sim 101$	$53 \sim 128$
#Outputs		2	2	2
#SPAN blocks		4	4	4
#SPAN units		$\{386, 386, 270, 270\}$	$\{371, 371, 260, 260\}$ $\sim \{1443, 1443, 1010, 1010\}$	$\{757, 757, 530, 530\}$ $\sim \{1829, 1829, 1280, 1280\}$
#Epochs		300	300	300
Batch size		128	128	128
Learning rate		$5 \times e - 3$	$5 \times e - 5$	$5 \times e - 5$
Decay rate		$5 \times e - 5$	$5 \times e - 7$	$5 \times e - 7$
Regularization		$3.1416 \times e - 2$	$3.1416 \times e - 2$	$3.1416 \times e - 2$
SPAN activation		SELU	SELU	SELU
Output activation	Prediction	Linear	Linear	Linear
	Classification	Sigmoid	Sigmoid	Sigmoid

FC: fully connected; SELU: Scaled Exponential Linear Unit

where $(+)$ is a classifier of positive samples, and $(-)$ is a classifier of negative samples.

The c-index score (CI) computes the ability to correctly provide a reliable ranking of the conversion times based on the individual risk scores.

$$\text{CI} = \frac{\sum_{i,j} 1_{T_j < T_i} \cdot 1_{f_j > f_i}}{\sum_{i,j} 1_{T_j < T_i}} \tag{S7}$$

where f_i is the risk score of a unit i, $1_{T_j<T_i} = 1$ if $T_j < T_i$ else 0, $1_{f_j>f_i} = 1$ if $f_j > f_i$ else 0.

The Brier score (BS) evaluates the accuracy of a predicted conversion function at a given time.

$$\text{BS}(t) = \frac{1}{n} \sum_{i=1}^{n} \left(\frac{\left(0 - \hat{S}(t, x_i)\right)^2 \cdot 1_{T_i \le t, E_i = 1}}{\hat{G}(T_i)} + \frac{\left(1 - \hat{S}(t, x_i)\right)^2 \cdot 1_{T_i > t}}{\hat{G}(t)} \right) \tag{S8}$$

where n is the number of samples, (x_i, E_i, T_i) are the datapoint, occurred event, and conversion time of sample i-th, $\hat{S}(t, x_i)$ is predicted conversion function, $\hat{G}(t)$ is the estomator of the conditional conversion function of the censoring times t calculated using the Kaplan-Meier method, $1_{T_i \le t, E_i=1} = 1$ if $T_i < t$ and $E_i = 1$ else 0, and $1_{T_i>t} = 1$ if $T_i > t$ else 0.

The mean absolute error (MAE) calculates the error rate between predicted values and true conversion time.

$$\text{MAE} = \frac{\sum_{i=1}^{n} \left| s_i - \hat{s}_{(i|p_i \ge 0.8)} \right|}{n} \tag{S9}$$

where s_i is the true conversion time of sample i-th, and $\hat{s}_{(i|p_i \ge 0.8)}$ is the predicted conversion time of sample i-th where the conversion probability $p_i \ge 0.8$, the threshold 0.8 was determined based on experiments.

ABLATION STUDIES

Performance on Combination of Radiomics Features

In this study, we extracted two types of radiomics characteristics which are shape and texture features from three brain regions: GM, WM, and CSF. Therefore, we conducted twenty-one combinations of different brain areas and radiomics types to determine the optimal combination. Evaluations of the time-to-AD prediction task (in c-index) and classification task (in accuracy) on each combination are demonstrated in Fig. S3 and Fig. S4, respectively. As can be observed from these figures, the usage of CSF resulted in the best performance on both tasks. In particular, we obtained c-index scores of 0.651, 0.672, and 0.661 for predicting time-to-AD conversion task when using combinations of $[CSF]$ $[shape]$, $[CSF]$ $[texture]$, and $[CSF]$ $[shape, texture]$, respectively. Otherwise, we also obtained the worse performance with using $[shape]$ features of the $[WM]$ region (achieved a c-index of 0.591) and of the $[GM]$ region (achieved a c-index of 0.61). When applying the $[texture]$ features of both regions, we obtained better c-index scores of 0.626 for $[GM]$ and 0.644 for $[WM]$. For the task of MCI-stage classification, we achieved low accuracy with using the $[shape]$ features over all the three regions, from 67.9% to 73.4%. The top-3 combinations are $[WM, CSF]$ $[texture]$, $[WM, CSF]$ $[shape, texture]$, and $[CSF]$ $[texture]$ with best accuracy of 79%, 78.8%, and 78.1%, respectively. Note that as CSF can be a powerful and accurate predictor of MCI patients who progressed to AD versus MCI patients who remained stable according to the findings in [2–4] and the $[shape]$ features is a good estimator for predicting time-to-AD conversion while the $[texture]$ features is good for classifying MCI-stage.

Performance using Hippocampus Subfields

In recent, several studies have highlighted the significance of utilizing hippocampal subfields for predicting the progression of Alzheimer's disease (AD). Kwak et al. [5] employed deep learning techniques to differentiate stable and progressive mild cognitive impairment (MCI) based on hippocampal morphometry, revealing specific subfields like presubiculum, CA1, subiculum, and molecular layer that played crucial roles in the prediction. Uysal et al. [6] focused on early AD diagnosis through neuroimaging analysis, specifically emphasizing the volumetric reduction in the hippocampus as a key indicator. They successfully distinguished between AD, MCI, and cognitively normal individuals using machine learning techniques based on hippocampal volume data. Sarasua et al. [7] compared different representations of the hippocampus and their impact on AD prediction using deep learning. They found that the choice of representation significantly influenced performance and interpretability. Overall, these findings emphasize the importance of considering hippocampal subfields in predicting AD progression accurately.

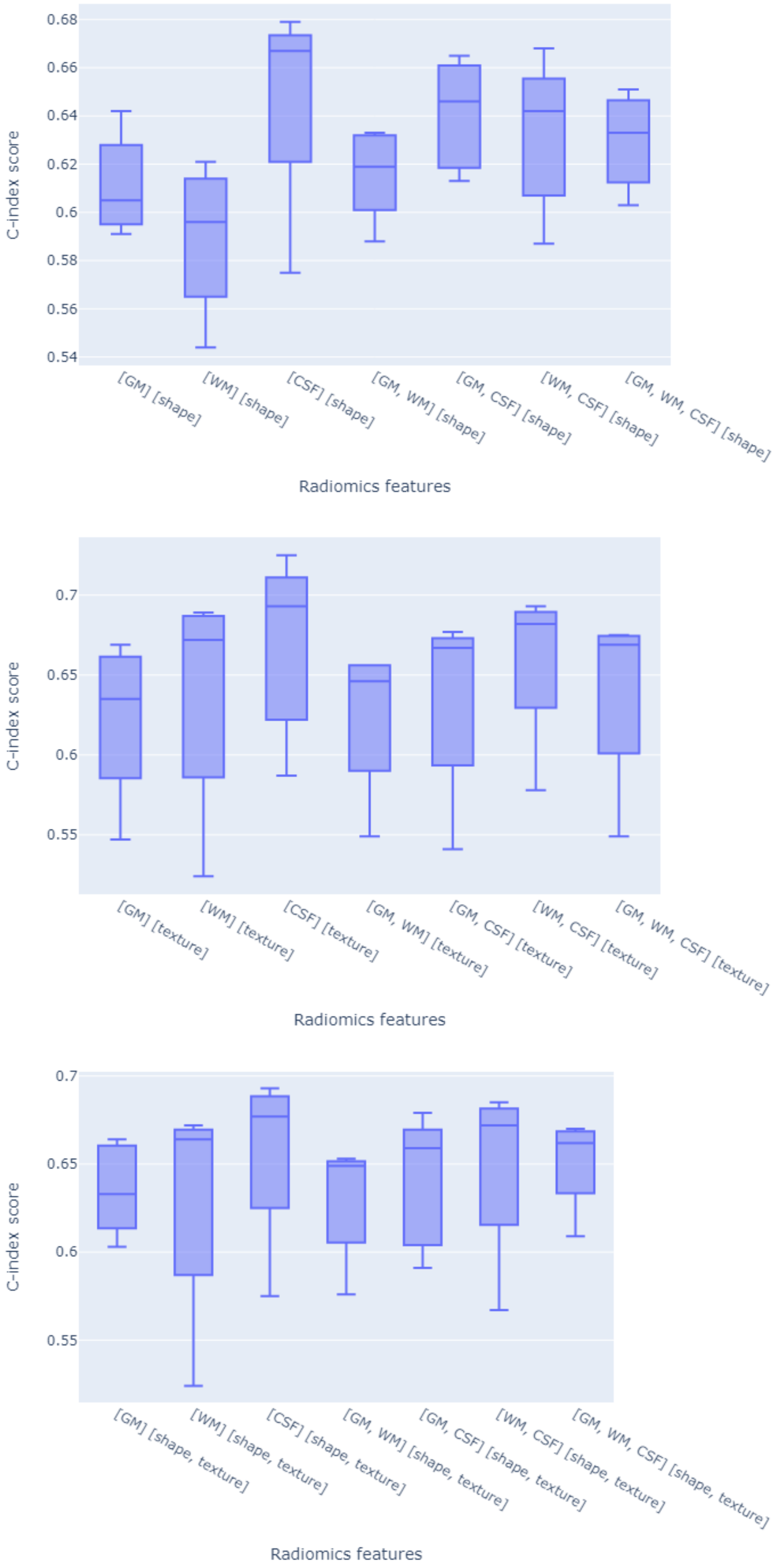

Fig. S3. Performance on task of time-to-AD prediction using a combination of radiomics features.

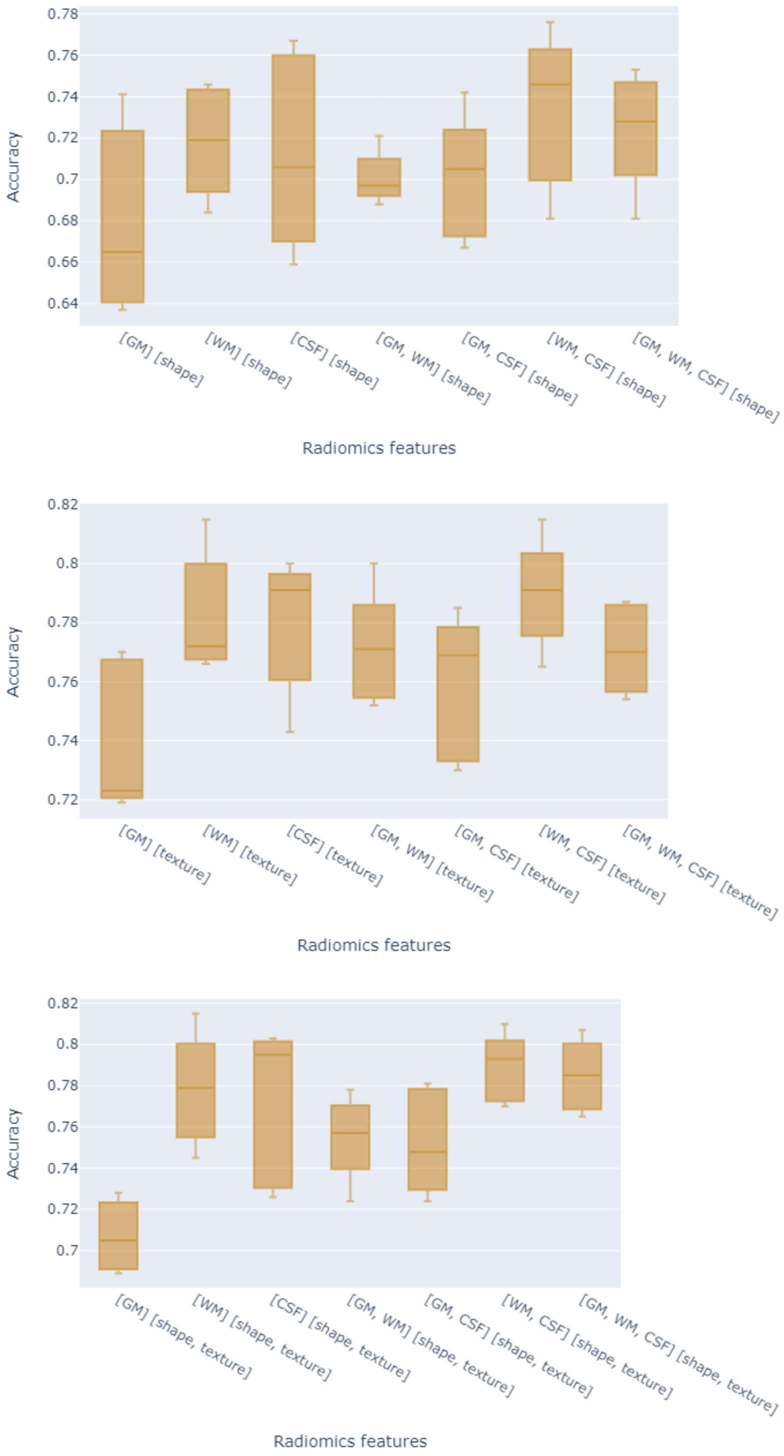

Fig. S4. Performance on classification task using a combination of radiomics features.

Table S5. Comparison of hippocampal features versus top-5 combination of radiomics.

Modality		Prediction Criteria			Classification Criteria		
		CI	BS	MAE	Acc	F1	AUC
Radiomics	$[CSF]$ $[shape]$	0.65	0.21	**545**	72.06	69.95	0.806
	$[CSF]$ $[texture]$	**0.67**	**0.19**	669	78.1	78.13	0.855
	$[CSF]$ $[shape, texture]$	0.66	0.19	882	76.04	74.89	0.794
	$[WM, CSF]$ $[texture]$	0.65	0.19	825	**79**	**78.53**	**0.856**
	$[WM, CSF]$ $[shape, texture]$	0.65	0.19	889	78.8	78.36	0.853
Hippocampus		0.64	0.22	673	73.52	70.11	0.762

Table S6. Comparison of multimodal approach using hippocampal and radiomics features combining with clinical data.

Multimodal approach	Prediction Criteria			Classification Criteria		
	CI	BS	MAE	Acc	F1	AUC
Radiomics + Clinical	**0.85**	**0.13**	**347**	**83.19**	**82.47**	**0.91**
Hippocampus + Clinical	0.81	0.16	403	82.35	80.85	0.9

In order to compare the utilization of hippocampus regions with radiomics, we obtained hippocampal features through the FreeSurfer V7 toolbox (https://surfer.nmr.mgh.harvard.edu/fswiki/DownloadAndInstall) and performed experiments to classify eMCI versus lMCI and predict the time-to-AD conversion using our proposed model. In total, we obtain 44 features related to hippocampal subregions. For comparison, we selected the top five performing combinations of radiomics features, namely $[CSF]$ $[shape]$, $[CSF]$ $[texture]$, $[CSF]$ $[shape, texture]$, $[WM, CSF]$ $[texture]$, and $[WM, CSF]$ $[shape, texture]$. Table S5 presents a comparison of two tasks for predicting AD progression for using hippocampal subfields compared to combinations of radiomics. The results show that the highest CI value of 0.67 was achieved using radiomics with CSF and texture features, indicating relatively good concordance in predicting time-to-AD conversion. However, the hippocampus-based approach achieved a slightly lower CI value of 0.64. In terms of classification performance, the radiomics approach showed higher accuracy (up to 79%) and F1 scores (up to 78.53%) compared to using the hippocampus alone. The AUC values, which measure the overall classification performance, were also higher for the radiomics approach. However, the hippocampus-based approach still demonstrated reasonable classification performance with an accuracy of 73.52%, F1 score of 70.11%, and AUC of 76.19%.

Table S6 presents a comparison between a multimodal approach combining hippocampus and radiomics features with clinical data for predicting AD progression. When combining radiomics features with clinical data, the multimodal approach achieved a CI value of 0.85, indicating a strong concordance in predicting time-to-AD conversion. This outperformed the hippocampus-based approach, which achieved a slightly lower CI value of 0.81. The Brier Score and MAE were also lower for the proposed approach, indicating improved accuracy and precision in predicting time-to-AD conversion. In terms of classification performance, the multimodal approach combining radiomics and clinical data achieved an accuracy of 83.19%, F_1 score of 82.47%, and AUC of 0.91 for distinguishing between early MCI and late MCI. The hippocampus-based approach combined with clinical data also demonstrated strong classification performance, with an accuracy of 82.35%, F_1 score of 80.85%, and AUC of 0.9.

Overall, we chose to use SPM (Statistical Parametric Mapping) and PyRadiomics tools for feature extraction due to several reasons. First, the extraction of hippocampal features using more extensive toolboxes like Freesurfer can be time-consuming, often taking around 5 hours per sample. In contrast, using SPM and PyRadiomics allowed us to extract features of gray matter, white matter, and cerebrospinal fluid (CSF) texture within approximately 15 minutes per sample. This time efficiency enabled us to process a larger dataset and perform more comprehensive anal-

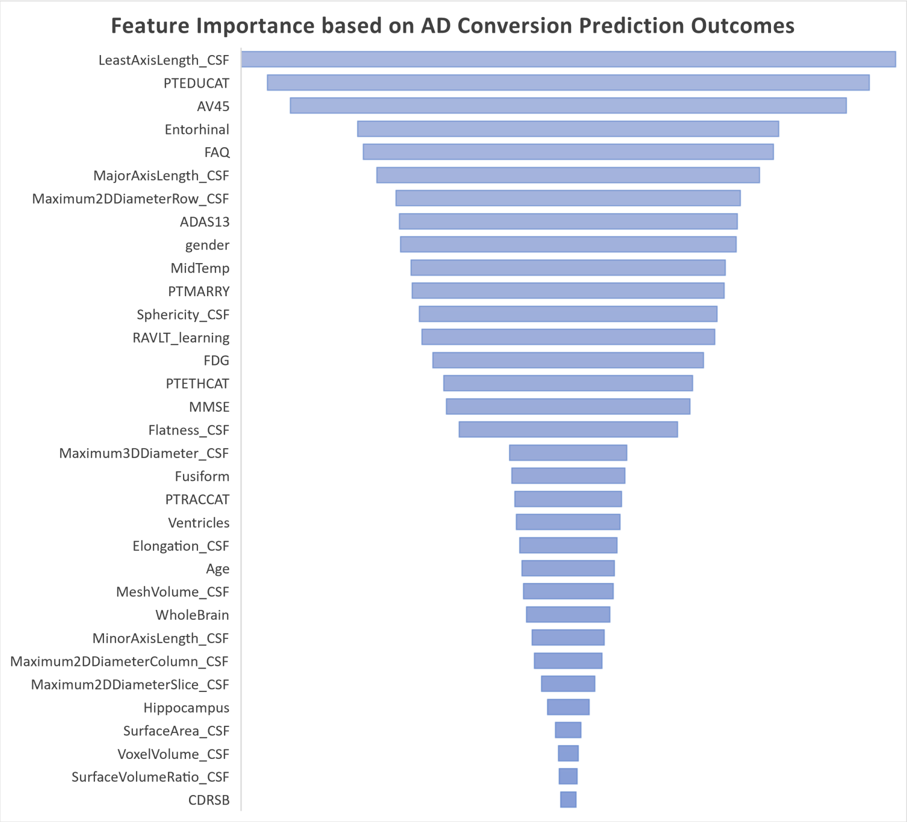

Fig. S5. Visualization of feature importance for AD conversion prediction.

yses. Second, by including features from gray matter, white matter, and CSF texture, we aimed to conduct a comparative analysis of the different brain regions, investigating their potential as biomarkers for AD. This approach allowed us to evaluate the performance and discriminatory power of various brain regions, including the hippocampus, in a comprehensive manner. Finally, even thought the hippocampus-based approach still shows promise in providing valuable predictive information, the results suggest that incorporating radiomics features, particularly CSF and texture features, enhances the performance of predicting AD progression compared to relying solely on hippocampal subregions.

Visualization of Feature Importance

Fig. S6 presents the visualization of feature importance based on specific outcomes. For the AD survival prediction task, the top-10 importance features are PTEDUCAT, AV45, ENTORHINAL volume, FAG ADAS13, GENDER, MIDDLE TEMPORAL volume of clinical features, and LEASTAXISLENGTH_CSF, MAJORAXISLENGTH_CSF, MAXIMUM2DDIAMETERROW_CSF of radiomic eatures. Meanwhile, for the MCI-state classification task, the top-10 featurues are MMSE, MIDDLE TEMPORAL volume, AGE of clinical data, and MAXIMUM2DDIAMETERROW_CSF, SURFACEAREA_CSF, SPHERICITY_CSF, SURFACEVOLUMERATIO_CSF, MAJORAXISLENGTH_CSF, ELONGATION_CSF, MESHVOLUME_CSF of radiomic data.

Conversion-time-to-AD Analysis

For the conversion-time-to-AD prediction task, we visualize the predicted conversion functions for various conditions to gain a more in-depth understanding of the influence of the proposed model, as seen in Fig. S7. In general, there are higher probabilities of conversion-time-to-AD conversion for the eMCI patients (average $P(T > t) > 70\%$) than the lMCI patients, as shown in Fig. S7a and S7b. In addition, people over the age of 70 have a higher risk of developing AD than younger patients, seen Fig. F9-c-f. Fig. F.9g and F.9h show that there is no substantial variation in AD conversion rates between males and females. From Fig. F.9i-k, it can be clearly seen that

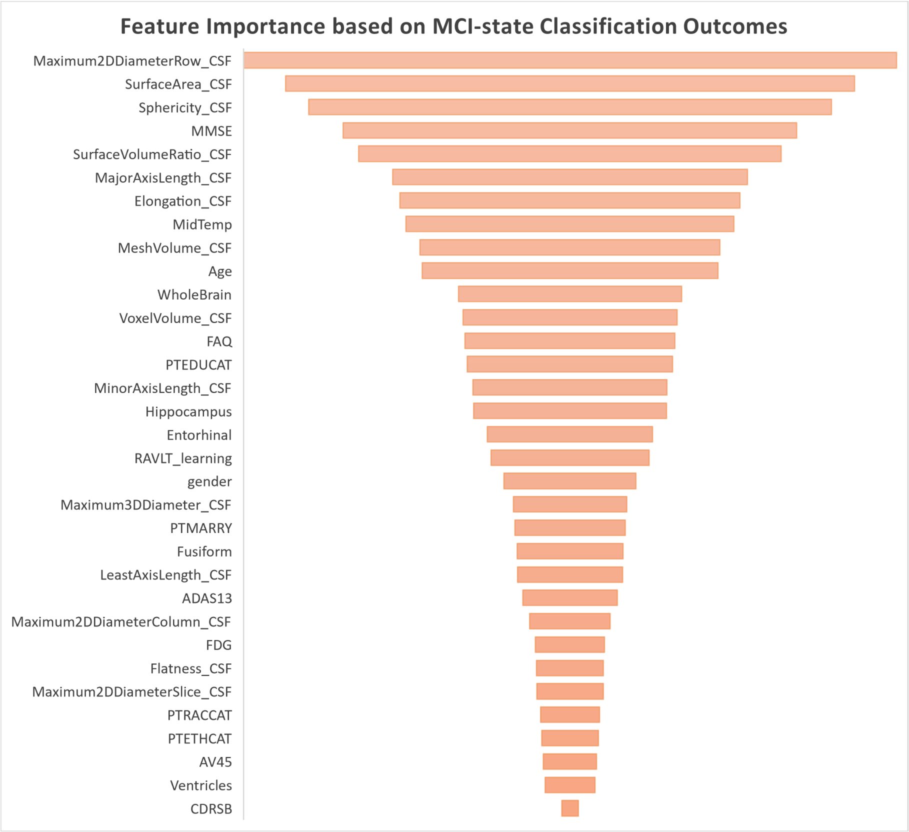

Fig. S6. Visualization of feature importance for MCI-state classification.

the higher the MMSE score, the lower the probability of AD conversion. There is less disparity across education groups, defined as those with a degree of education from 8 to 15 and more than 15 years, as shown in Fig. F.9l and F.9m. In the ethnicity property, there are two categories: Hispanic/Latino and Not Hospanic/Latino., however, only one patient has an unknown ethnicity (seen Fig. F.9p). Basically, the Hispanic/Latino group (Fig. F.9n) has a greater conversion rate than the other one (Fig. F.9o). Furthermore, those who never marry or are divorced have similar conversion rates, whereas those who marry or are widowed have a higher possibility of AD conversion, as seen in Fig. F.9q-t. Finally, we look at the race property, which indicates that black individuals (Fig. F.9v) have a higher likelihood of surviving than both Asian (Fig. F.9u) and white people (Fig. F.9w).

Fig. S7. Conversion time prediction for specific cases. (a) eMCI patients. (b) lMCI patients. (c) Patients who are younger than 65. (d) Patients who are between 65 and 70. (e) Patients who are in 80s. (f) Patients who are in 90s. (g) Male patients. (h) Female patients. (i) Patients whose MMSE score in range $24-26$. (j) Patients whose MMSE score in range $27-28$. (k) Patients whose MMSE score in range $29-30$. (l) Patients whose education level in range $8-15$. (m) Patients whose education level greater than 15. (n) Patients whose ethnicities are Hispanic and Latino. (o) Patients whose ethnicities are not Hispanic nor Latino. (p) Patient who is unknown ethnicity. (q) Patients who never married. (r) Patients who married. (s) Patients who widowed. (t) Patients who divorced. (u) Patients who are asian race. (v) Patients who are black race. (w) Patients who are white race.

See

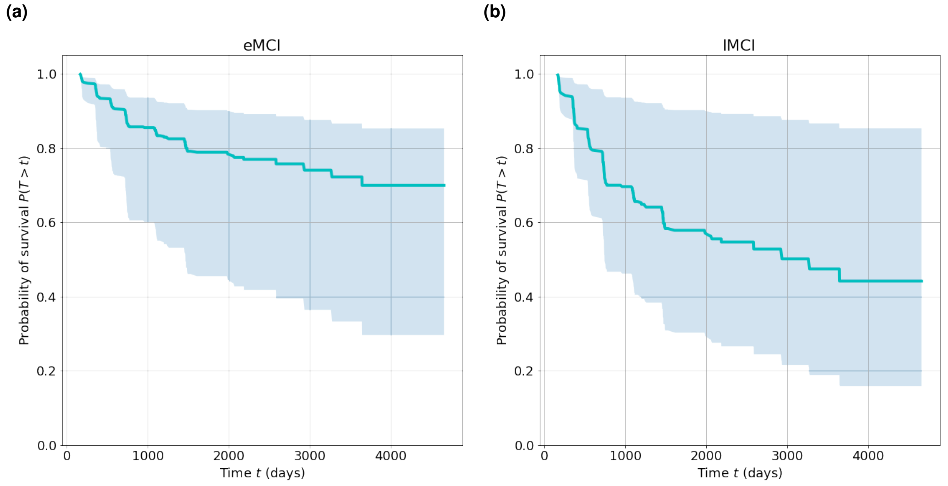

the next page...

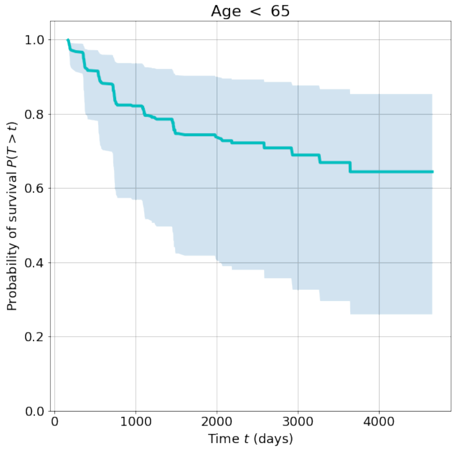

(c)

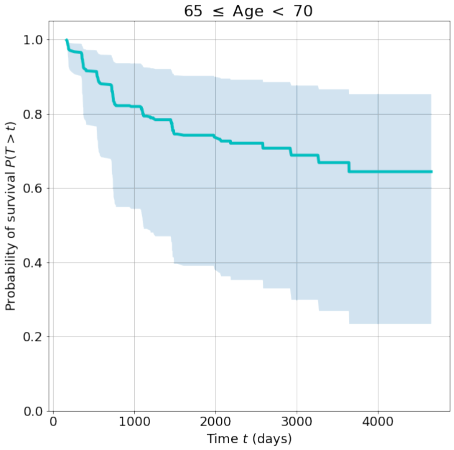

(d)

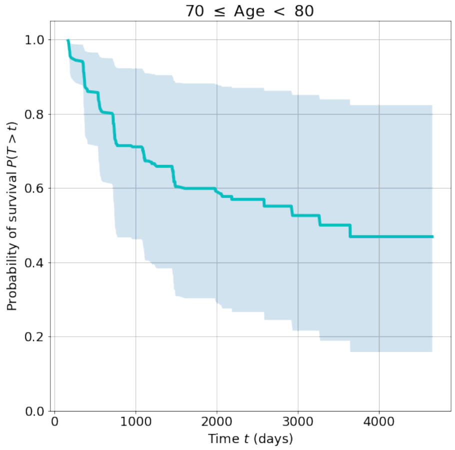

(e)

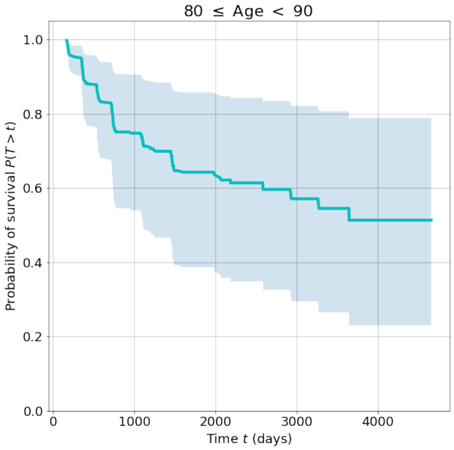

(f)

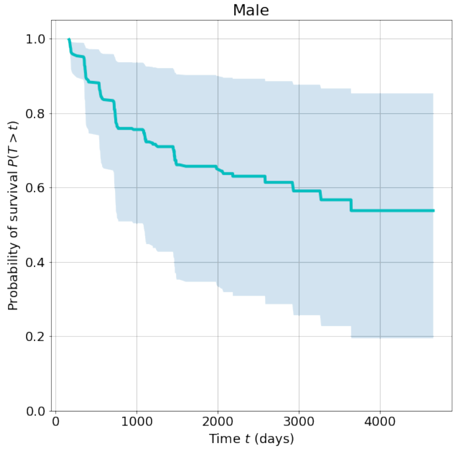

(g)

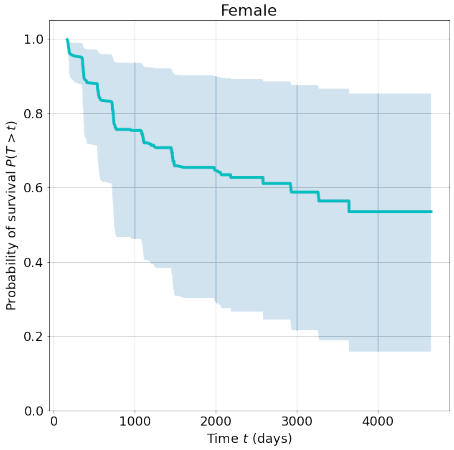

(h)

See the next page...

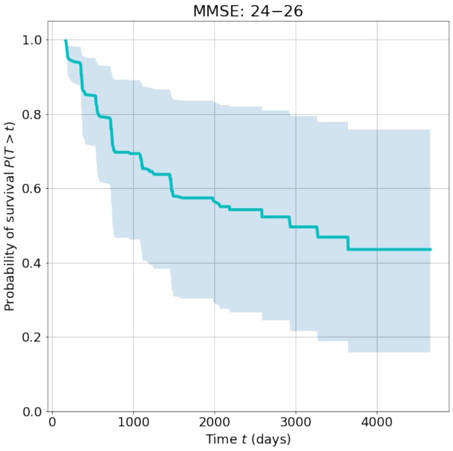

(i)

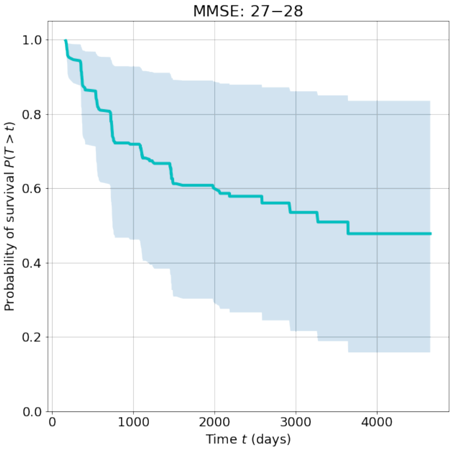

(j)

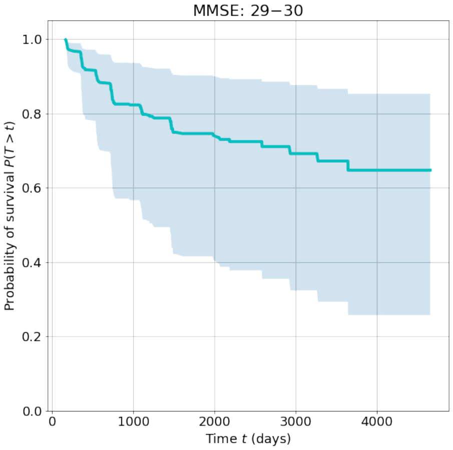

(k)

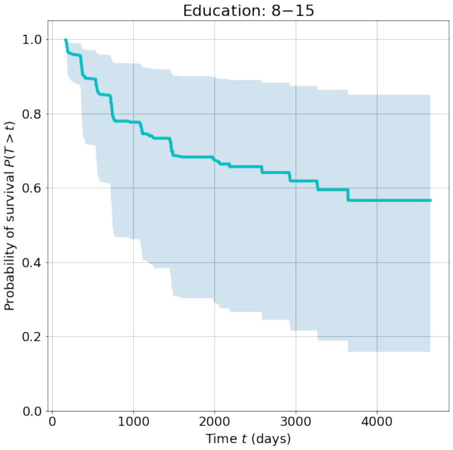

(l)

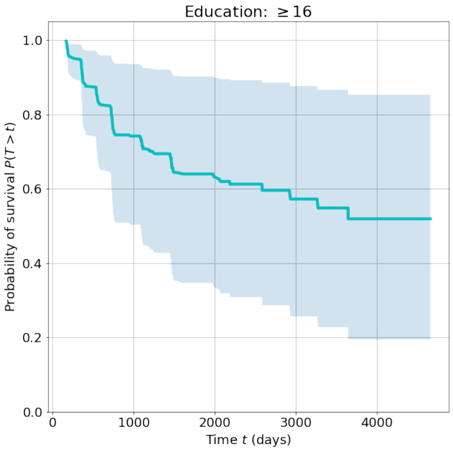

(m)

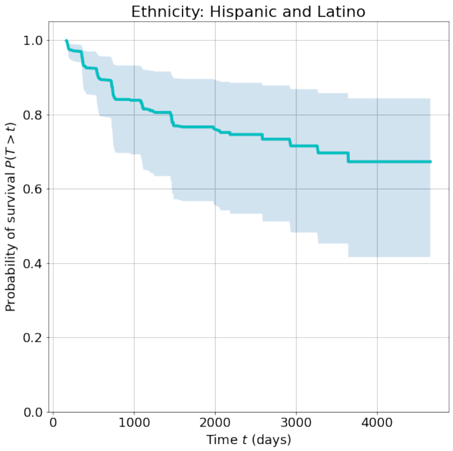

(n)

See the next page...

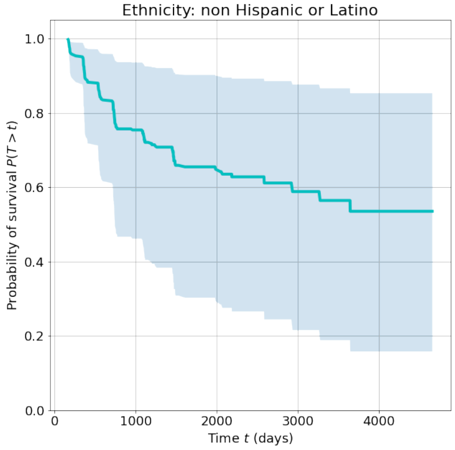

(o)

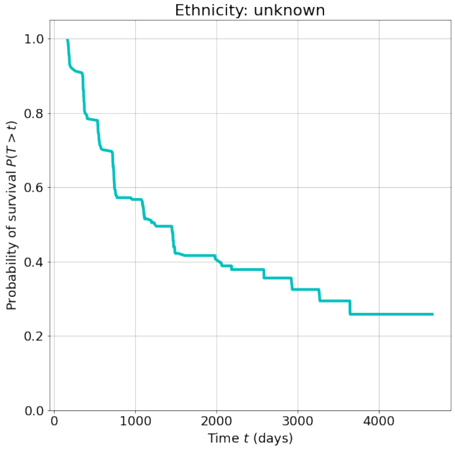

(p)

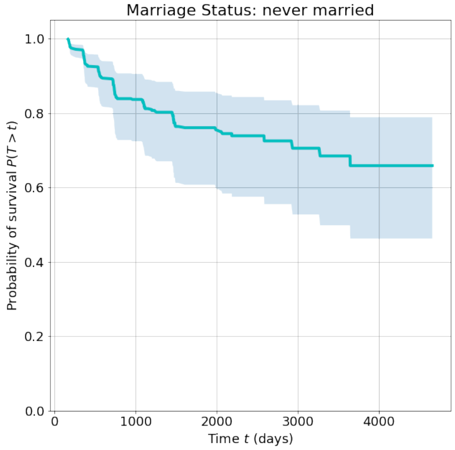

(q)

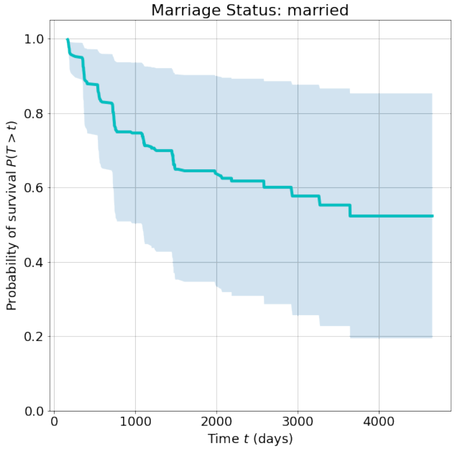

(r)

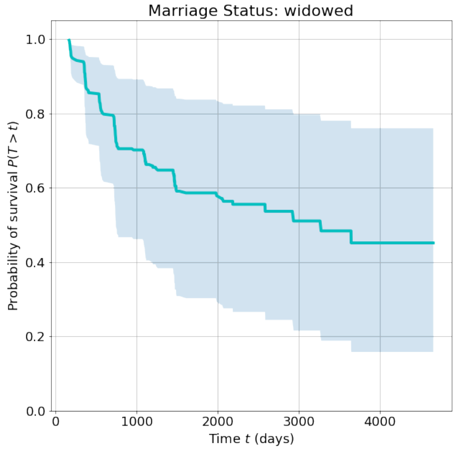

(s)

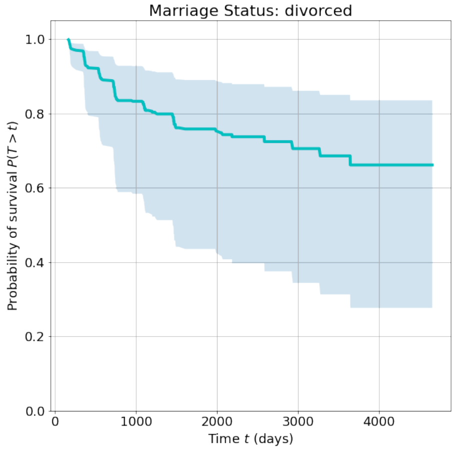

(t)

See the next page...

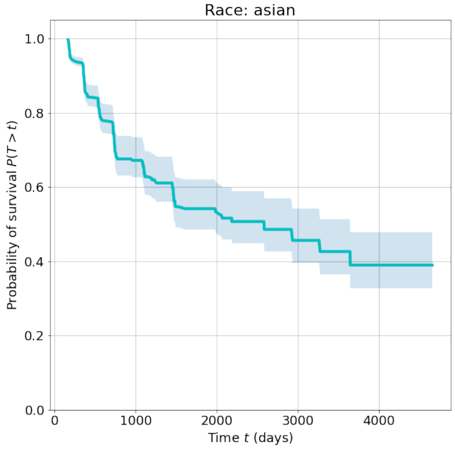

(u)

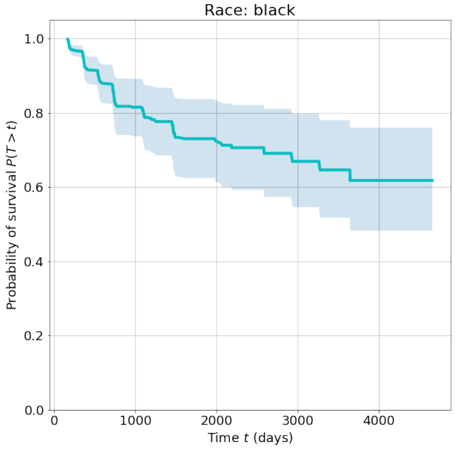

(v)

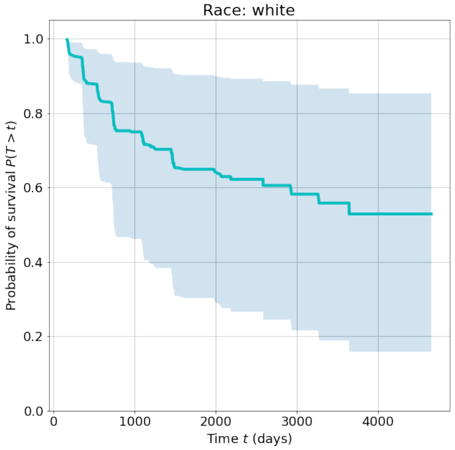

(w)

REFERENCES

1. D. P. Kingma and J. Ba, "Adam: A method for stochastic optimization," arXiv preprint arXiv:1412.6980 (2014).
2. L. Parnetti, A. Lanari, G. Silvestrelli, E. Saggese, and P. Reboldi, "Diagnosing prodromal alzheimer's disease: role of csf biochemical markers," Mech. ageing development **127**, 129–132 (2006).
3. M. Brys, E. Pirraglia, K. Rich, S. Rolstad, L. Mosconi, R. Switalski, L. Glodzik-Sobanska, S. De Santi, R. Zinkowski, P. Mehta *et al.*, "Prediction and longitudinal study of csf biomarkers in mild cognitive impairment," Neurobiol. aging **30**, 682–690 (2009).
4. S. J. Vos, B. A. Gordon, Y. Su, P. J. Visser, D. M. Holtzman, J. C. Morris, A. M. Fagan, and T. L. Benzinger, "Nia-aa staging of preclinical alzheimer disease: discordance and concordance of csf and imaging biomarkers," Neurobiol. aging **44**, 1–8 (2016).
5. K. Kwak, M. Niethammer, K. S. Giovanello, M. Styner, E. Dayan, A. D. N. Initiative *et al.*, "Differential role for hippocampal subfields in alzheimer's disease progression revealed with deep learning," Cereb. Cortex **32**, 467–478 (2022).
6. G. Uysal and M. Ozturk, "Hippocampal atrophy based alzheimer's disease diagnosis via machine learning methods," J. Neurosci. Methods **337**, 108669 (2020).
7. I. Sarasua, S. Pölsterl, and C. Wachinger, "Hippocampal representations for deep learning on alzheimer's disease," Sci. reports **12**, 8619 (2022).
